# Fractionation of a sex-inducing substance from flatworms using open-column chromatography and reverse-phase high-performance liquid chromatography

**DOI:** 10.1016/j.xpro.2023.102625

**Published:** 2023-10-27

**Authors:** Kiyono Sekii, Taro Watanabe, Riku Ito, Akitoshi Yoshikawa, Madoka Ichikawa-Seki, Kimitoshi Sakamoto, Kazuya Kobayashi

**Affiliations:** 1 Faculty of Agriculture and Life Science, Hirosaki University, Bunkyo-cho 3, Hirosaki, Aomori 036-8561, Japan; 2 Faculty of Business and Commerce, Keio University, Hiyoshi 4-1-1, Yokohama, Kanagawa 223-8521, Japan; 3 Laboratory of Veterinary Parasitology, Faculty of Agriculture, Iwate University, 3-18-8 Ueda, Morioka, Iwate 020-8550, Japan

**Keywords:** Developmental Biology, Biotechnology and Bioengineering, Chemistry

## Abstract

A substance that sexualizes planarians, an ancestral group of parasitic flatworms, is widely present in planarians and parasitic flatworms. Here, we present a protocol for extracting and purifying the active fraction with sex-inducing activity. We describe steps for homogenization of flatworms, sample concentration, open-column chromatography, and reverse-phase high-performance liquid chromatography. We then detail a feeding bioassay to confirm sex-inducing activity. The obtained active fraction may positively affect parasitic flatworm sexual maturation and can be tested by adding it into the culture media.

For complete details on the use and execution of this protocol, please refer to Sekii et al. (2023).^1^

## Highlights

• Protocol for obtaining sex-inducing substance from planarians and parasitic flatworms
• Fr. M30 is produced by open-column chromatography of homogenized sexual flatworms
• Fr. M30 is the active fraction and can be further fractionated using HPLC
• The sex-inducing activity of the obtained fraction is confirmed by a feeding bioassay

## Abstract (editor highlights)

Publisher’s note: Undertaking any experimental protocol requires adherence to local institutional guidelines for laboratory safety and ethics.

## Graphical abstract

**Figure:**
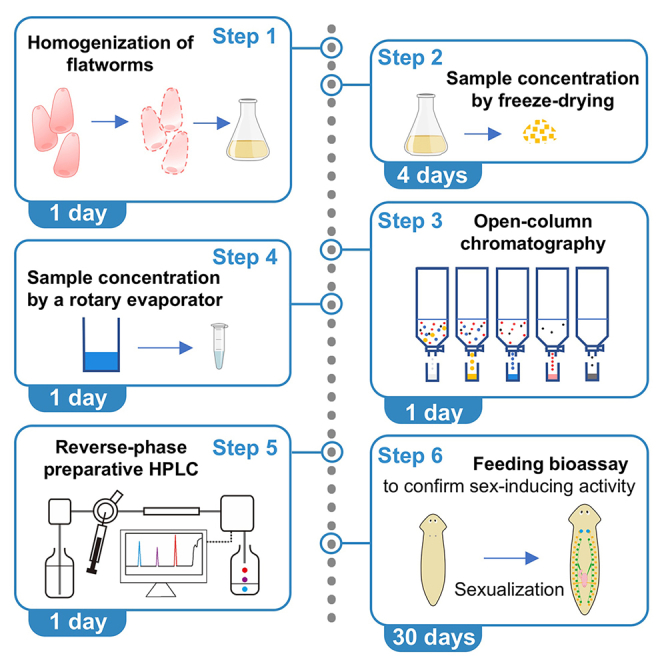

## Before you begin

A sex-inducing substance can induce sexual maturation in planarians, which is the evolutionary ancestors of parasitic flatworms,^2^^,^^3^ and promote a reproductive mode switch from the asexual to sexual state. During this sexualization process, germ cells and hermaphroditic reproductive organs are newly formed from pluripotent stem cells present throughout the planarian’s body. Although the molecular identity of the sex-inducing substance is still unknown, a recent study found that the sex-inducing substance is widely present in planarians and parasitic flatworms (monogeneans and flukes).^1^ The role of the sex-inducing substance in parasitic flatworms is still unknown. However, it is highly possible that the sexual maturation system triggered by the sex-inducing substance also exists in parasitic flatworms, since similarities in the stem cell system between the planarians and their parasitic relatives have been reported in previous studies.^3^^,^^4^^,^^5^^,^^6^^,^^7^^,^^8^^,^^9^ The sexual maturation process of parasitic worms in the host body is still largely unknown. However, the addition of the fraction containing a sex-inducing substance, obtained by this protocol, to the culture medium may enable us to observe their sexual maturation *in vitro* and to elucidate the role of the sex-inducing substance in parasitic flatworms.

The protocol below describes the specific steps for extracting the sex-inducing substance from the parasitic flatworm, the fluke *Calicophoron calicophorum*. However, we have also used this protocol for the terrestrial planarian *Bipalium nobile* and believe that the protocol can be applied to a wide range of flatworms, from planarians to parasitic groups.

There are several points to note about the flatworms used for the extraction of the sex-inducing substance. (1) Use species with a yolk gland system, as the sex-inducing substance is produced and/or stored in the yolk glands.^1^^,^^10^ (2) Use sexually mature worms; the free-living (non-parasitic) flatworms should be collected in their breeding season, and the parasitic flatworms should be in a sexually reproducing state. (3) Among parasitic flatworms, the monogeneans and flukes contain the sex-inducing substance; however, tapeworms do not [at least when the extraction is attempted with the amount of worms used in this protocol (8 g wet weight)]. To check the technical aspects of this protocol, the planarian and parasitic flatworm used in studies by Nakagawa et al. (2018),^10^ Sekii et al. (2023),^1^ and others^11^^,^^12^ could serve as positive controls, since they are known to contain the sex-inducing substance.

As stated in the protocol, the presence of a sex-inducing substance in the extracted samples can be ascertained by feeding them to the OH strain, an asexual clonal population of the planarian *Dugesia ryukyuensis*, and observing whether they undergo sexual differentiation. The OH strain of *D. ryukyuensis* will be provided upon request. We strongly recommend using the OH strain for the following reasons: (1) The OH strain never spontaneously sexualized in 40 years when kept at 20°C, and (2) the OH strain can be stably sexualized by chemical stimulation with the sex-inducing substance, making it a suitable test organism for evaluating the activity of the extracted fraction.

The most reliable way to verify the presence of the sex-inducing substance in the flatworms of interest is to assess whether the OH strain becomes sexualized by directly feeding them the flatworms of interest, prior to performing a large-scale purification. In this case, (1) perform a feeding bioassay as described in this protocol; feed asexual worms of the OH strain with the flatworms of interest instead of the freeze-dried chicken liver homogenate. (2) Keep the flatworms of interest frozen, and scrape off a small amount with a knife at feeding time. (3) The amount of food should be a few millimeters square that is eaten in 7–10 min each day, according to the size of growing worms.

### Collection of flatworms for the extraction

**Timing: it depends on the cases**

1. Collect and store 8 g (wet weight) of the flatworms of interest for extraction at −80°C until use.

***Note:*** Flatworms are not for sale and cannot be ordered. The readers need to collect their own sexual flatworms that contain the sex-inducing substance (i.e., meeting the criteria mentioned in the “before you begin” section) by themselves. For example, in the studies by Nakagawa & Sekii et al. (2018)^10^ and Sekii et al. (2023),^1^ various sexual flatworms were obtained by propagation in the laboratory (e.g., the *Fasciola hybrid* and *Schistosoma mansoni* flukes), collection in the field during breeding season (e.g., the *Bipalium nobile* and *Bdellocephala brunnea* planarians), and extraction from animals infected with parasitic flatworms at veterinary clinics and farms. To collect the *C. calicophorum* fluke used in this protocol, we obtained the fourth stomachs of infected cows from a slaughterhouse and extracted the worms from them with tweezers.

***Note:*** Before freezing, remove excess water to the maximum extent possible without damaging the tissue.

### Preparation of test worms for feeding bioassays

**Timing: 3 weeks**

**Figure 1:**
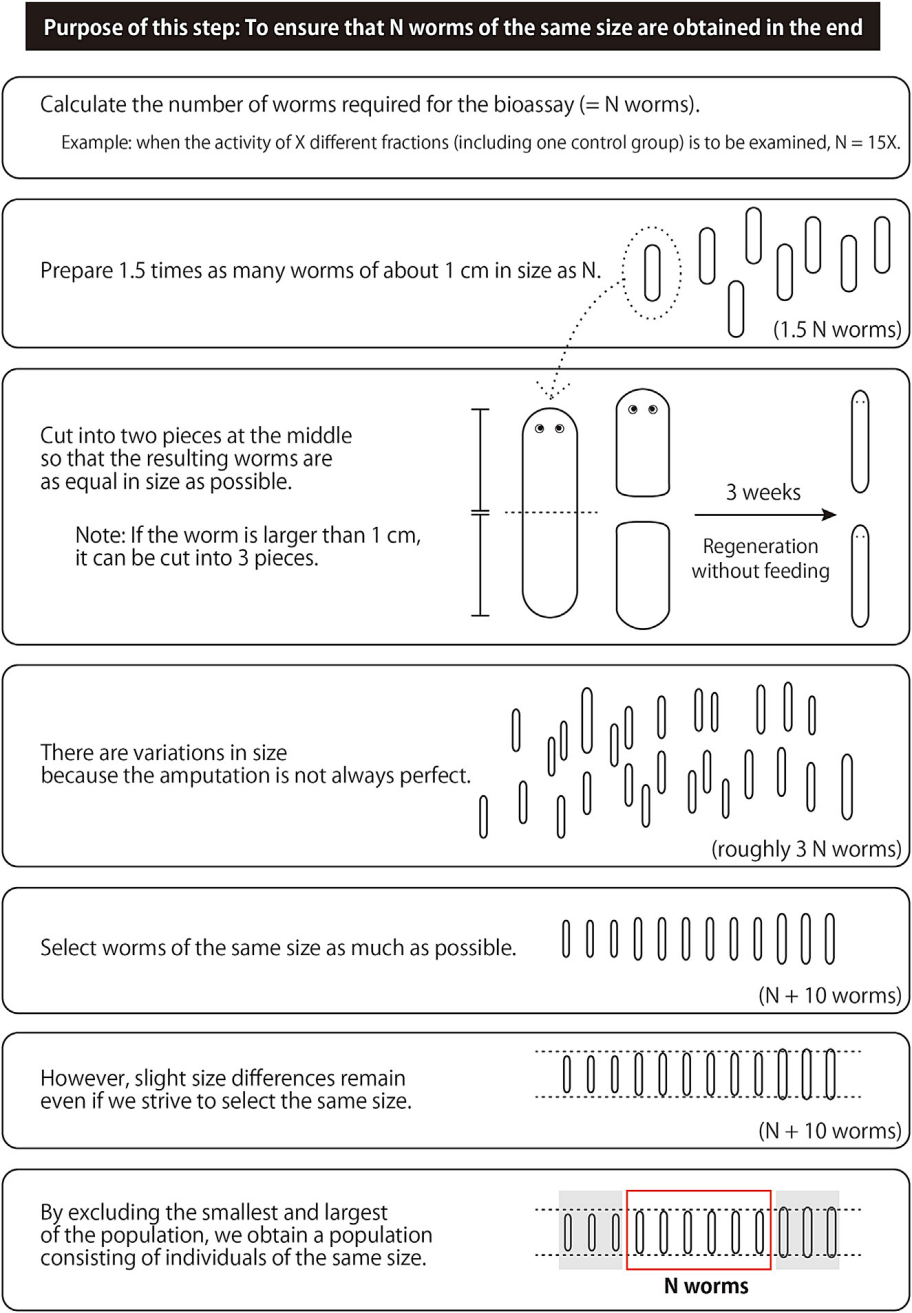
Summary of how to prepare the test worms for feeding bioassay

For the feeding bioassay to evaluate sex-inducing activity, we recommend using the OH strain of the planarian *D. ryukyuensis*. For the bioassay, small worms ranging 5–6 mm in length (starved) with uniform size should be used. Such test worms are prepared by amputating worms larger than or equal to 1 cm and letting them regenerate without feeding (starved) for 3 weeks as shown in Figure 1. (1) The most important thing is preparing small starved worms that are equal in size for the feeding bioassay. (2) Worms are transversely cut at the middle of their bodies to obtain resulting pieces that are as equal in size as possible. (3) As cuttings made by humans are not always perfect, there will be variation in the size of worms after regeneration and starvation (Figure 1). (4) In anticipation of this, the approach selected by our protocol is to prepare three times the number of worms needed and selecting worms of the same size among them (Figure 1).

***Note:*** Worms larger than or equal to 1 cm are ONLY used for cutting and regeneration to reach a final length ranging 5–6 mm.

***Note:*** If the feeding bioassay is begun with larger worms, they will divide (transverse fission) during the assay period due to body growth, so small worms should be prepared; asexual reproduction (transverse fission) ceases in the later stages of sexualization in *D. ryukyuensis*.^13^ Moreover, since sexualization is promoted by providing a certain amount of the sex-inducing substance, the use of small worms can increase the amount of the daily sex-inducing substance provided per body weight.

***Note:*** Cutting should take place 3–4 days after the last feeding. The day after feeding should be particularly avoided.

Unless specified, all steps should be performed at 20°C.

**Figure 2:**
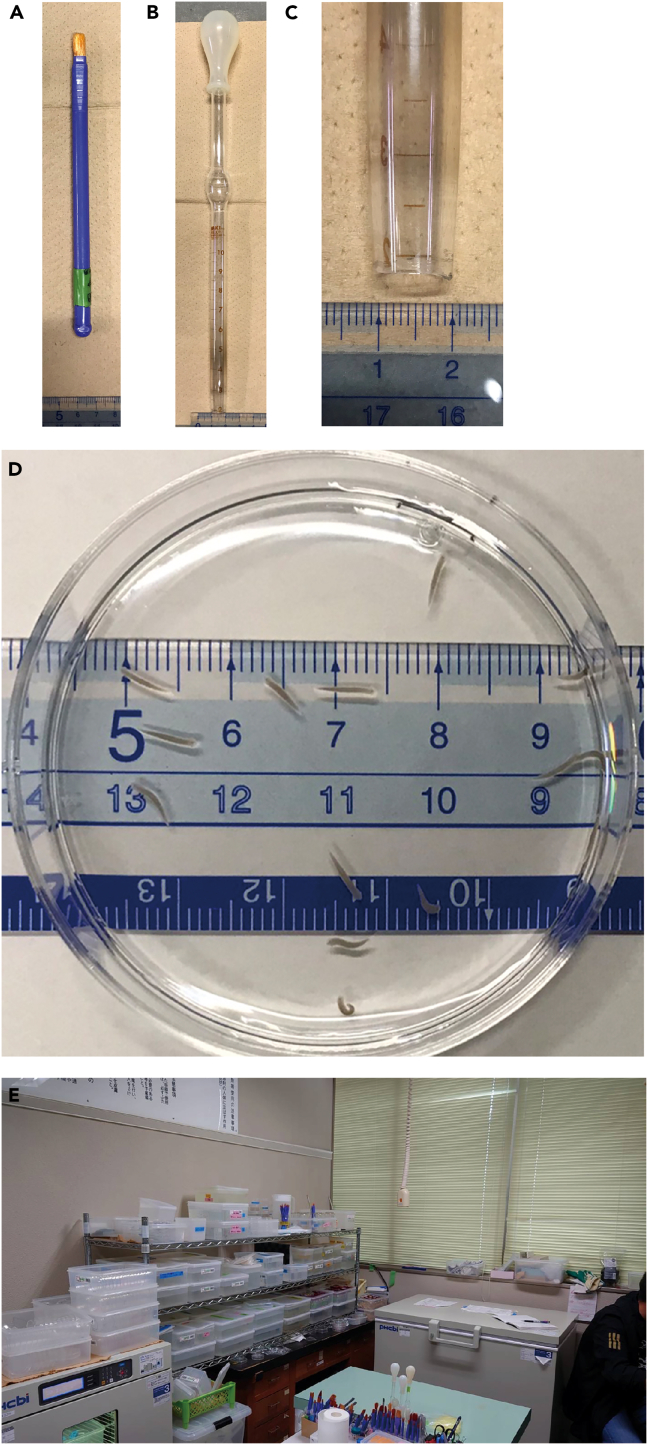
Materials required to prepare the test worms for feeding bioassay (A) A paintbrush for handling planarians in shallow or low water environments (e.g., plastic petri dish). (B) A wide-mouth (inner tip diameter of about 1 cm) 10-mL glass pipette for handling planarians in environments with a certain depth of water (e.g., Tupperware). (C) Image showing the diameter of the wide-mouth glass pipette tip. (D) Example of worms about 5–6 mm in length. (E) Room for maintaining the planarians.
  b. Wide-mouth (inner tip diameter of about 1 cm) 10-mL glass pipette (Figures 2B and 2C): Used when handling planarians in environments with a certain depth of water (e.g., Tupperware). If one needs to prepare their own pipettes by cutting them, it is recommended to burn them with a burner to round off the cut end.

***Note:*** The point is to choose tools to handle the planarians gently without stressing them.

***Note:*** We recommend using plastic petri dishes, which can be disposed of after a certain period of use, because products that planarians come into contact with should not be washed with detergent.

2. Prepare rearing water to maintain planarians.
  a. Autoclave tap water and cool it at 20°C.
3. Prepare tools for handling planarians.
  a. Commonly available paintbrush (Figure 2A): Used when handling planarians in shallow or low water environments (e.g., plastic petri dish).

4. Select the required number of asexual worms, 1 cm or larger, suitable for amputation.
  a. Calculate the number of worms needed for the bioassay: 15 worms per group are used. When the sex-inducing activity of X different fractions (including one control group) is to be examined, 15X worms are needed.
  b. Select worms (1 cm or larger) so that the number of worms after amputation becomes at least three times the number required for the bioassay. The reason is to be able to select worms of uniform size from the regenerating population. Use the following table as appropriate.

***Note:*** Worms of approximately 1 cm in length should be amputated into two pieces and larger worms into three pieces. To ensure that the pieces are of equal size after cutting, the recommended cutting positions are in the middle if cutting into two pieces or at every third of body length if cutting into three pieces.

**Table:** Preparation of worms

The number of worms required for the bioassay	Number of worms to select in this step (worms 1 cm in length)	Number of worms to select in this step (worms larger than 1 cm in length)
30	45	30
45	68	45
60	90	60

5. Place a few worms on, for example, the lid of a 60 mm plastic petri dish with a paintbrush (Methods video S1) and amputate them into 2–3 pieces using a sharp razor blade (e.g., ultrathin carbon steel blade FA-10, FEATHER Safety Razor, Japan) so that they are as close to the same size as possible.

***Note:*** When cutting into three pieces, the first amputation should be made from the tail side so that the planaria will extend and the second amputation will be easier to make (Methods video S1).

6. Transfer the planarian fragments to a Tupperware container filled with rearing water by soaking (Methods video S1). A paintbrush can also be used to transfer the fragments, but should be done carefully to avoid damaging them.
7. Change the rearing water 2–3 h after cutting.

**CRITICAL:** Be sure to change the rearing water because digestive enzymes leaking from the amputated fragments will deteriorate the water quality and planarian condition. If the pharynx is observed to spit out, it should be removed as it can also cause spoilage.

8. Maintain the planarian fragments for 3 weeks without feeding. Change the rearing water once a day for the first 2 days and thereafter once a week. When handling them, be careful not to damage them until regeneration is complete.
9. On the day before or the day of the start of the bioassay (step 64), select worms by matching their size.
  a. Select the required number of worms plus 10 worms about 5–6 mm in length (Figure 2D) and place them in one container. For example, when testing X groups (including one control group), select 15X + 10 worms.
  b. Check the size of the selected worms, and remove 10 worms in total that are the smallest or largest in the population (Figure 1).
  c. Divide the worms into X groups of 15 worms each.
  d. Start the bioassay.

**CRITICAL:** Worms should be as equal in size as possible. Variation in size will result in variation in feeding among individuals within a group (e.g., monopolization by larger ones), making it difficult to perform a suitable bioassay.

***Note:*** Usual maintenance of the OH strain is as follows (planarians not used for bioassays should be reared in this manner). (1) Environment: Keep worms at 20°C in a container such as a Tupperware container filled with rearing water (tap water autoclaved and cooled to 20°C) in an air-conditioned room (20°C) (Figure 2E). Neither CO_2_ nor any other type of gas is used; changing the composition of the atmosphere is unnecessary. No specific light cycle conditions are needed. (2) Feeding: Give a piece of beef or chicken liver (the size of a fingertip is enough) once a week. Liver can be frozen and no homogenization is needed. Feed worms until they leave the liver (about 1–3 h), and change the rearing water as follows. (3) Change of rearing water: Once a week. Usually, a rearing water change after feeding is sufficient. Scrub the inside of the container with a brush to remove dirt (mucus, food scraps, excrement, etc.). Detergent should not be used.

**CRITICAL:** A survey of 665 sites across Japan reported that the mean hardness of Japanese tap water was 48.9 ± 25.8 (1σ SD) and the median was 46.0 mg/L.^14^ The hardness of available tap water may need to be adjusted accordingly for animal care.

Methods video S1. How to cut the planarians, related to steps 4 and 5 in the “before you begin” section

## Key resources table

**Table:** 

REAGENT or RESOURCE	SOURCE	IDENTIFIER
**Chemicals, peptides, and recombinant proteins**		

Cosmosil 75 C18–OPN	Nacalai Tesque (Japan)	Cat#37842-95
MeOH for washing ODS gel [EP (Extra Pure reagent) grade]	Nacalai Tesque (Japan)	Cat#21914-74
MeOH (HPLC grade)	Nacalai Tesque (Japan)	Cat#21929-23
MeCN (HPLC grade)	Nacalai Tesque (Japan)	Cat#00430-83
Milli-Q water (HPLC grade)	Merck Millipore (USA)	N/A
l-Tryptophan	Nacalai Tesque (Japan)	Cat#35607-74
Ethylene glycol as refrigerant [EP (Extra Pure reagent) grade]	Nacalai Tesque (Japan)	Cat#15208-53

**Experimental models: Organisms/strains**		

*D. ryukyuensis* (the OH strain, adult, asexual)	Hirosaki University	N/A

**Software and algorithms**		

ChromNAV version 2	JASCO (Japan)	N/A

**Other**		

0.5 mL microcentrifuge tube	Greiner Bio-One (Austria)	Cat#667201
1.5 mL microcentrifuge tube	Greiner Bio-One (Austria)	Cat#616201
2 mL microcentrifuge tube	Sarstedt (Germany)	Cat#72.695.500
25 mL conical centrifuge tube	IWAKI (Japan)	Cat#2362-025-MYP
50 mL conical centrifuge tube	Labcon (USA)	Cat#3181-345
P1000 micropipette for 100–1000 μL	Nichiryo (Japan)	Cat#00-NPX2-1000
P200 micropipette for 20–200 μL	Nichiryo (Japan)	Cat#00-NPX2-200
1000 μL micropipette tip	Nippon Genetics (Japan)	Cat#FG-402
200 μL micropipette tip	Nippon Genetics (Japan)	Cat#FG-301
500 mL beaker	N/A	N/A
500 mL glass bottle	N/A	N/A
2 L glass bottle	N/A	N/A
Plastic tray: Balance tray natural (10 mL)	AS ONE (Japan)	Cat#1-5233-01
Tweezers	N/A	N/A
Potter-type homogenizer set (10 mL)	AS ONE (Japan)	Cat#5-5721-03
Homogenizer: Homogenizer Agitator HK-1	AS ONE (Japan)	Cat#1-2050-11
Ultrasonic disruptor model UR-200P	Tomy Seiko (Japan)	N/A
Ultrasonic cleaner USC-100Z38S-22	IWAKI (Japan)	N/A
Centrifuge: Sorval Legend XFR	Thermo Fisher Scientific (USA)	Cat#75004540
Rotor for a centrifuge: Fiberlite F15-8 x 50cy	Thermo Fisher Scientific (USA)	Cat#75003663
Ultracentrifuge: Himac CP80NX	HITACHI Koki (Japan)	Cat#5720100810
Rotor for an ultracentrifuge: Fixed angle rotor P50AT2	HITACHI Koki (Japan)	Cat#5720211118
Tube for ultracentrifuge: Himac 33 PC thick-walled tube	HITACHI Koki (Japan)	Cat#338457A
Even balance	N/A	N/A
Nalgene Rapid-flow 0.2 μm PES filters	Nalge Nunc International (USA)	Cat#597-4520
Aspirator: MS-S	Kenis (Japan)	Cat#1-136-0750
Millex-GV syringe-driven Filter Unit, 0.22 μm, PVDF	Merck Millipore (USA)	Cat#SLGVR33RS
Millex-HV syringe-driven Filter Unit, 0.45 μm, PVDF	Merck Millipore (USA)	Cat#SLHVR33RS
TERUMO Disposable 1 mL syringe for filtartion	TERUMO (Japan)	Cat#SS-01T
TERUMO Disposable 10 mL syringe for filtartion	TERUMO (Japan)	Cat#SS-10SZ
Lyophilizer: FDU-1200	EYELA (Japan)	Cat#208880
Vacuum pump for a lyophilizer: GLD-136C	ULVAC (Japan)	Cat#A460_3000_0525
Bottle for lyophilizer (1200 mL)	EYELA (Japan)	Cat#64-4060-88
Medicinal powder papers	Hakuai (Japan)	Cat#2040
Rubber bands	N/A	N/A
TERUMO syringe needle 18G (1.20 mm × 38 mm)	TERUMO (Japan)	Cat#NN-1838S
Econo-column (50 φ× 500 mm)	Bio-Rad (USA)	Cat#7375051
Column stand	N/A	N/A
Peristaltic pump: MP-2000	EYELA (Japan)	Cat#251280
Spatula	N/A	N/A
Long Pasteur pipette	IWAKI (Japan)	Cat#IK-PAS-9P
Wide-mouth PP bottle Eyeboy (250 mL)	AS ONE (Japan)	Cat#5-002-03
Wide-mouth PP bottle Eyeboy (500 mL)	AS ONE (Japan)	Cat#5-002-04
Rotary evaporator: N-1300V	EYELA (Japan)	Cat#266430
Water bath for a rotary evaporator: SB-350	EYELA (Japan)	Cat#180180
Chiller for a rotary evaporator: CCA-1112A	EYELA (Japan)	Cat#268450
Vacuum pump for a rotary evaporator: NVP-1000	EYELA (Japan)	Cat#261800
Receiving flask (1 L) for a rotary evaporator	EYELA (Japan)	Cat#116340
Trap ball for a rotary evaporator	EYELA (Japan)	Cat#116800
200 mL eggplant-shaped flask	N/A	N/A
10 mL pear-shaped flask	N/A	N/A
Paint brush	Pentel (Japan)	Cat#XZBNF-6
10 mL glass pipette	Maruemu (Japan)	Cat#0801-05
2 mL glass pipette	Maruemu (Japan)	Cat#0805-02
Organic chicken liver	Champool (Japan)	N/A
90 mm plastic Petri dish	N/A	N/A
35 mm plastic Petri dish	N/A	N/A
60 mm plastic Petri dish	N/A	N/A
Ultrathin carbon steel blade FA-10	FEATHER Safety Razor (Japan)	N/A
Luer-lock syringe	N/A	N/A
Syringe for cleaning	N/A	N/A
Sample syringe (2.5 mL): 2.5MDR-LC-GT	SGE (Australia)	Cat#008505
Syringe filter for HPLC: mdi 0.2 μm syringe Filter, PVDF	Advanced Microdevices (India)	Cat#SYVF0301MNXX104
HPLC column: Develosil C30-UG-5 (20 φ × 250 mm)	Nomura Chemical (Japan)	Cat#UG175P2250W
PU-2089 plus Quaternary gradient pump	JASCO (Japan)	N/A
UV-2075 plus Intelligent UV detector	JASCO (Japan)	N/A
Interface: LC-Net II/ADC data collector	JASCO (Japan)	N/A
External degasser: BG-42-10	FLOM (Japan)	Cat#SGA-G3005
1 mL sample loop: RHEODYNE Stainless steel loop for 7725i	Rheodyne (USA)	Cat#7755-027
Flow cell for UV detector: TP cell (optical path length 10 mm)	JASCO (Japan)	Cat#6824-H110A
Aluminum foil	N/A	N/A
Microscope: Olympus SZX10	Olympus (Japan)	N/A
Flexible arm light	N/A	N/A

## Materials and equipment

• Rearing water: autoclave tap water and cool at 20°C.
• 10% aqueous MeOH solution: add 30 mL MeOH to 270 mL Milli-Q water (total 300 mL).
• 30% aqueous MeOH solution: add 90 mL MeOH to 210 mL Milli-Q water (total 300 mL).
• 50% aqueous MeOH solution: add 150 mL MeOH to 150 mL Milli-Q water (total 300 mL).

This solution cannot be stored because MeOH volatilization changes the concentration.

**CRITICAL:** MeOH is critical reagent and there is no alternative. It may be possible to extract the sex-inducing substance using solvents other than MeOH, but we have not tested how efficient this extraction would be compared to using MeOH.

• 10% aqueous MeCN solution: add 180 mL MeCN in 1620 mL Milli-Q water (total 1800 mL).

Prepare in a 2 L glass bottle. This solution cannot be stored because MeCN volatilization changes the concentration.

• Tryptophan solution (1.33 mg/mL): add 20 mg tryptophan to 15 mL Milli-Q water.

Store at 4°C. Maximum storage time is 3 months.

***Note:*** Use HPLC (high-performance liquid chromatography)-grade Milli-Q water (LC-Pak), MeOH, and MeCN.

**CRITICAL:** MeOH and MeCN should be handled in a fume hood and appropriate safety gloves and safety goggles should be worn. Collect and dispose the waste liquid appropriately.

***Alternatives:*** This protocol uses a Lyophilizer FDU-1200 (EYELA, Japan) and a vacuum pump GLD-136C (ULVAC, Japan), but any equivalent system can alternatively be used.

***Alternatives:*** This protocol uses a Rotary evaporator system N-1300V (EYELA, Japan), but any equivalent system can alternatively be used.

***Alternatives:*** This protocol uses an HPLC system (JASCO PU-2089 plus Quaternary gradient pump, JASCO UV-2075 plus Intelligent UV detector, JASCO LC-Net II/ADC data collector, and a software ChromNAV version 2, JASCO, Japan), but any equivalent system can alternatively be used.

## Step-by-step method details

### Extraction of supernatant containing the sex-inducing substance from flatworms (Day 1)

**Timing: 1 day**

**Figure 3:**
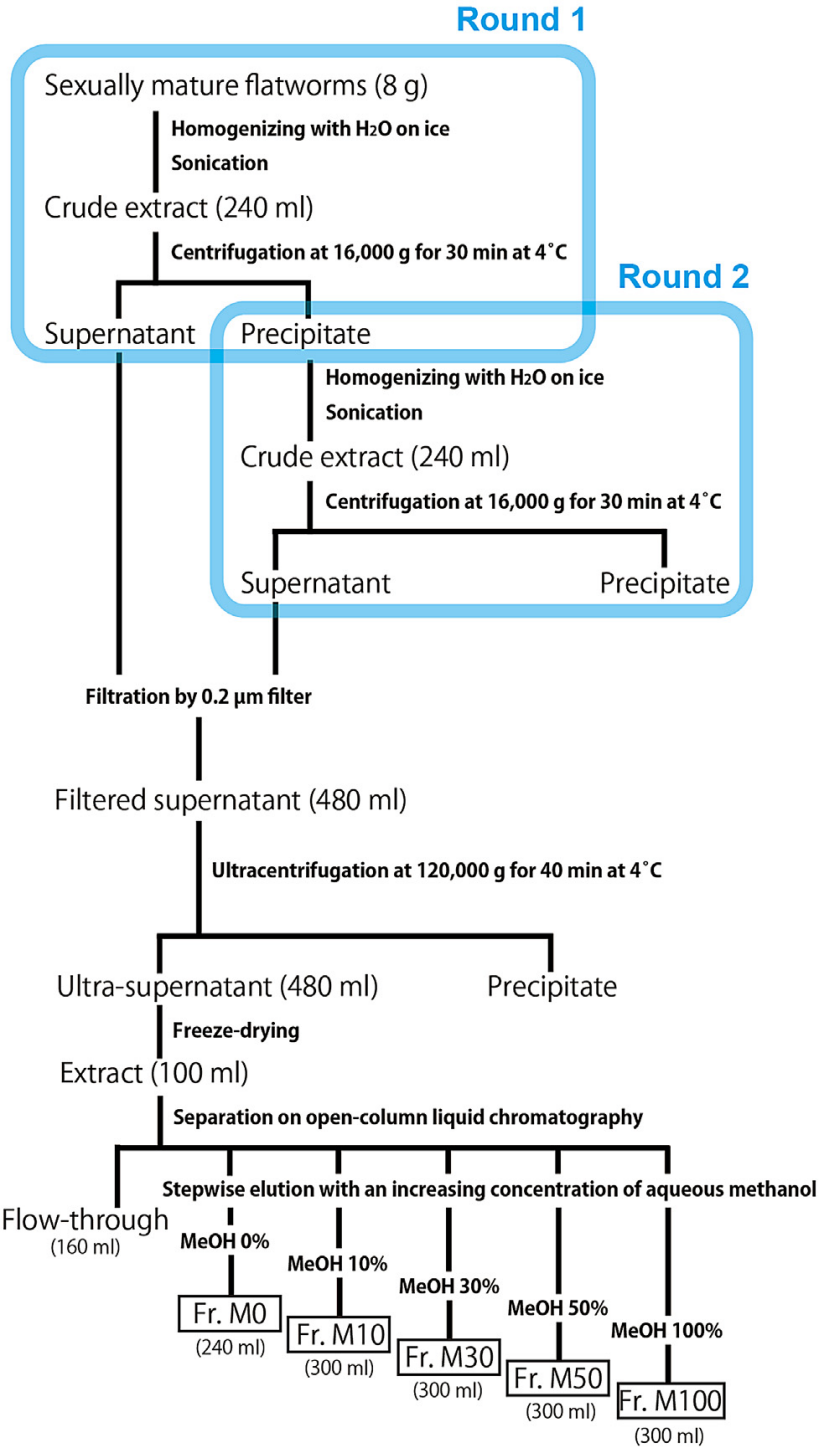
Summary of how to prepare Frs. M0-M100

The flatworm of interest is homogenized, and the supernatant is collected by centrifugation. The sex-inducing activity is mainly present in the supernatant. A schematic flow of this step is shown in Figure 3.

Unless specified, all steps should be performed at 4°C or on ice.

1. Prepare 50 mL conical centrifuge tubes as follows.

**Table:** Preparation of tubes

Labeling	Number of 50 mL tubes
“Homogenized”	8
“Supernatant: 1^st^ round”	8
“Supernatant: 2^nd^ round”	8
“Ultra-supernatant”	14 (it depends on the capacity of the ultracentrifuge machine)

**Figure 4:**
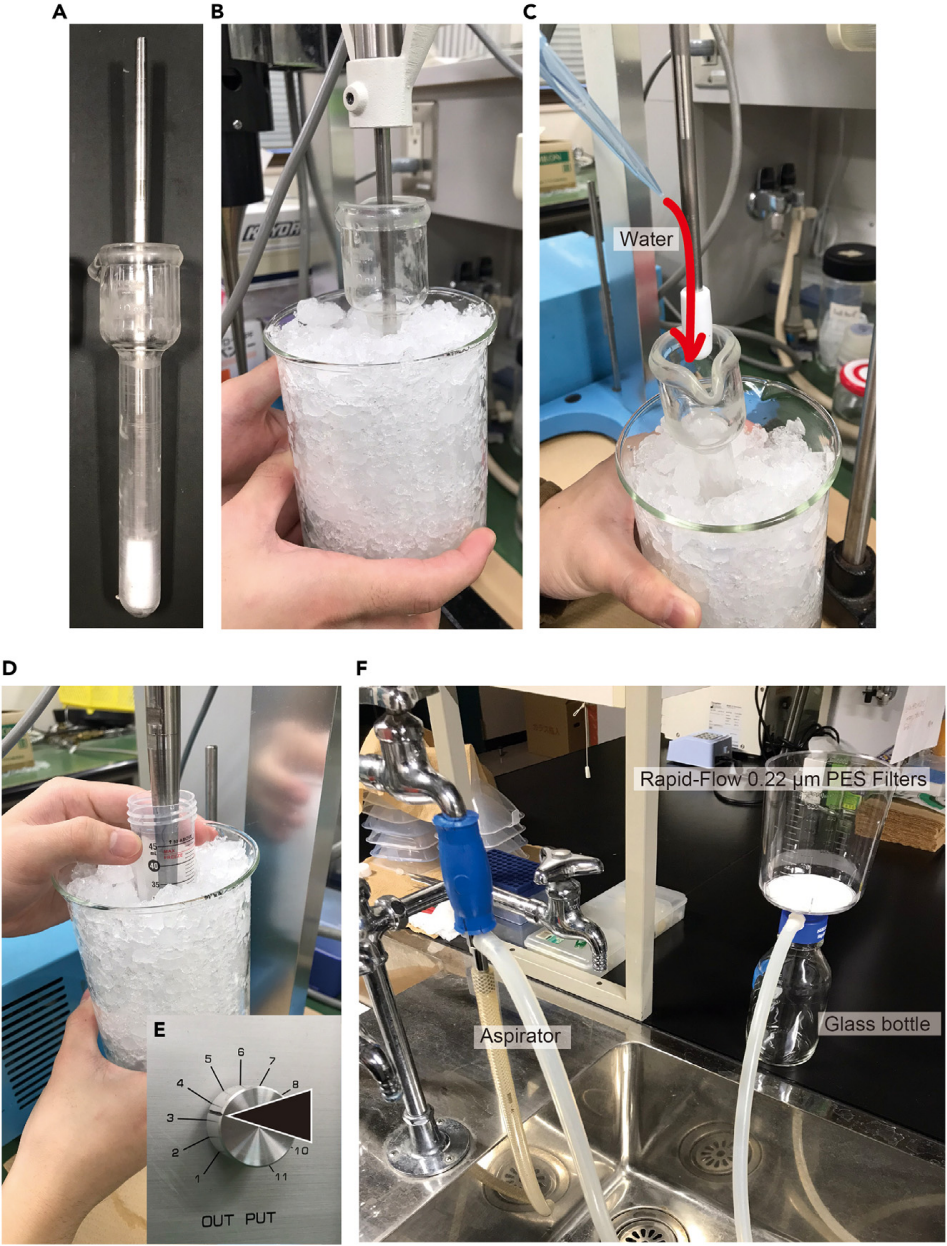
Extraction of flatworms (A) A Potter-type 10 mL homogenizer. (B) Homogenization while cooling with ice. (C) How to rinse the homogenizer (including the rod) with Milli-Q water. (D) Sonication while cooling with ice. (E) Image of the output dial showing the ultrasonic intensity. (F) Sample filtration system using NALGENE Rapid-Flow 0.2 μm PES Filters (Nalge Nunc International).
3. Sonicate the homogenized sample.
  a. Sonicate continuously for 2 min 30 s, while moving the sample tube evenly (Figure 4D; Methods video S3).
    ***Note:*** For ultrasonic intensity, about 30% of the maximum output of a typical ultrasonic device would be appropriate. For example, in our device (Ultrasonic disruptor Model UR-200P, TOMY Seiko, Japan), we set the output dial to 3.5 out of 11 levels (Figure 4E).
    ***Note:*** Work while cooling with ice as heat is generated (Figure 4D).
    **CRITICAL:** The tube will be damaged by prolonged contact with the ultrasonic device in the same place, leaking fluid.
    Methods video S3. How to sonicate, related to step 3
  b. Keep the samples on ice until the next step (step 4).
4. Centrifugation and supernatant collection.
  a. Cool the centrifuge and rotor to 4°C prior to centrifugation.
  b. Balance the weight of tubes.
    ***Note:*** Add Milli-Q water little by little to balance the tubes.
  c. Centrifuge at 16,000 g (4°C) for 30 min (at speed).
  d. Transfer the supernatant to 50 mL conical centrifuge tubes.
    i. If it is the first round (Figure 3), transfer to the tubes labeled with "Supernatant: 1st round" by decantation.
    ii. If it is the second round (Figure 3), transfer to the tubes labeled with "Supernatant: 2nd round" by decantation; after decanting, collect the remaining sample using a P1000 micropipette.
  e. Keep the samples on ice until the next step (step 5).
5. To increase the efficiency of the extraction of the sex-inducing substance, the substance remaining in the precipitate that is not recovered in the supernatant is extracted by a second-round extraction.
  a. Repeat steps 2–4 with the precipitate instead of 1 g of flatworms.
    i. Add about 3 mL of Milli-Q water to the precipitate in the 50 mL tube and float the precipitate with a spatula.
    ii. Quickly decant the floating precipitate into a homogenizer.
      ***Note:*** Transfer the precipitate to the homogenization tube with a slight shaking motion. Without some momentum, only water will be transferred, and the precipitate will remain in the 50 mL tube.
    iii. Add an additional 2 mL of Milli-Q water to the 50 mL tube and collect as much of the remaining precipitate as possible.
    iv. Perform homogenization (see step 2) on the precipitate collected in a total of 5 mL of Milli-Q water.
    v. Return the homogenized sample to the same 50 mL tube and repeat sonication and centrifugation (see steps 3 and 4).
  b. Keep the samples on ice until the next step (step 6).
    ***Note:*** Using the planarian *Bdellocephala brunnea*, we confirmed that the second-round extracts also have sufficient sex-inducing activity (Kobayashi, unpublished data). The third-round extracts still have some activity, but we do not perform a third extraction to improve the working efficiency.
6. Sample filtration.
  a. Set up NALGENE Rapid-Flow 0.2 μm PES Filters (Nalge Nunc International) (Figure 4F) using, for example, a clean glass bottle.
  b. Perform filtration on all samples (total 480 mL). Both first- and second-round extracts can be mixed.
  c. Keep the samples on ice until the next step (step 7).

***Note:*** When the filter becomes clogged, replace it with a new one. We often use two for the fluke *C. calicophorum*.

2. Homogenize 8 g of the flatworm. We used, for example, a Potter-type 10 mL homogenizer (Figure 4A). Homogenization is done 1 g at a time and repeated eight times.
  a. Divide 8 g of flatworms evenly into eight portions, place 1 g each on a plastic tray, and store at −20°C until just before homogenizing.
  b. Transfer 1 g of flatworms with tweezers into the homogenizer.
  c. Add 5 mL of Milli-Q water.
    ***Note:*** Since the sex-inducing substance is apparently a papain-resistant low-molecular weight compound weighing less than 500 Da,^15^ we did not use any protease inhibitors during purification.
  d. Homogenize in a continuous mode until no fragments are visible. When no fragments are visible, repeat the strokes 20 more times (Methods video S2).
    ***Note:*** Work while cooling with ice as heat is generated (Figure 4B).
    ***Note:*** The intensity of homogenization should be varied depending on the hardness of the flatworms used; for example, the intensity should be adjusted so that flatworms of any hardness are homogenized at an average of about 3,000 rpm. In the case of *C. calicophorum*, for example, it takes approximately 4–5 s per stroke (round trip) and approximately 25 strokes before the worm fragments are no longer visible.
    Methods video S2. How to homogenize, related to step 2
  e. Transfer the sample to a 50 mL conical centrifuge tube labeled "Homogenized" by decantation. Also, rinse the homogenizer (including the rod) with Milli-Q water and collect the remaining sample (Figure 4C).
  f. Add Milli-Q water and fill up to 30 mL.
  g. Keep the samples on ice until the next step (step 3).

***Optional:*** If the above described filtration system is not available, a syringe (e.g., TERUMO Disposable 10 mL syringe, TERUMO, Japan) and filter (e.g., Millex-GV Syringe-driven 0.22 μm PVDF Filter Unit, Merck Millipore, USA) can be used. However, this is not recommended for the following reasons: (1) it requires a lot of force, (2) the solution passes through the filter very slowly, (3) the filter is easily clogged, resulting in a lot of labor and sample loss due to frequent filter changes, and (4) many filters need to be consumed, which is costly.

**Figure 5:**
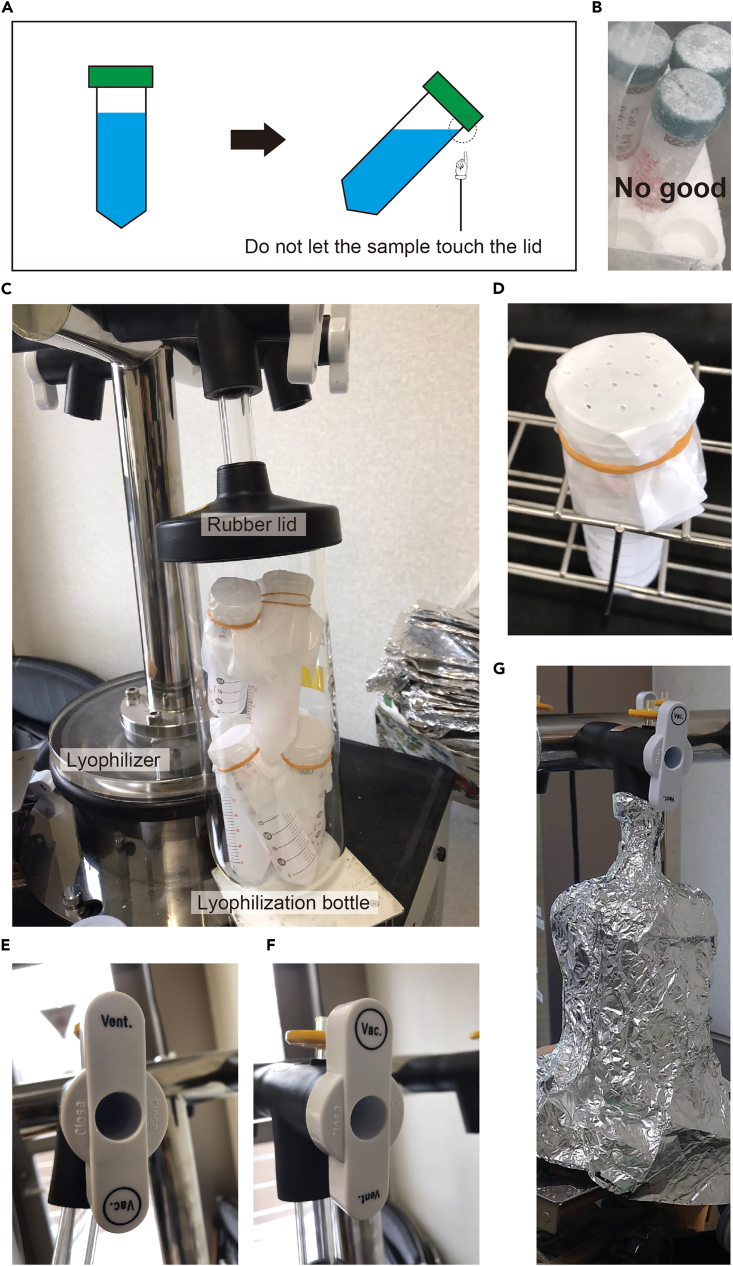
Freeze-drying of the flatworm extract (A) Freezing with tubes tilted to increase the surface area of the frozen sample. (B) Bad example: use of tube stands made of insulating material during freezing. (C) Freeze-drying. (D) Example of a sample tube with a lid made of medicinal powder paper with multiple needle holes. (E) Cock in the "ventilation" state. (F) Cock in the "vacuum" state. (G) Lyophilization bottle shielded from light by aluminium foil.

7. Ultracentrifugation and supernatant collection.
  a. Cool the rotor at 4°C, for example, by using a cold room or refrigerator, prior to ultracentrifugation.
    ***Note:*** The ultracentrifuge itself does not need to be specifically pre-cooled, since condensation caused by cooling would take extra time to depressurize.
  b. Transfer 15 mL of the filtered supernatant into each tube for ultracentrifugation.
    ***Note:*** The amount of sample that can be ultracentrifuged at one time depends on the centrifuge model available and should be adjusted accordingly.
  c. Balance the weight of the tubes.
    ***Note:*** Add Milli-Q water little by little to balance the tubes.
  d. Centrifuge at 120,000 g (4°C) for 40 min (at start).
    **CRITICAL:** Carefully handle the large ultracentrifuge. It can be very dangerous if used incorrectly. The balanced pair of the tubes must be placed on the diagonal of the rotor.
  e. Collect every last drop of supernatant carefully with a P1000 micropipette and transfer to 50 mL conical centrifuge tubes labeled "Ultra-supernatant".
    ***Note:*** Our ultracentrifuge is capable of running 15 mL × 12 tubes at a time, so the entire sample is ultracentrifuged three times, resulting in approximately 14 ″Ultra-supernatant" tubes.
  f. When about 30–40 mL of the sample has accumulated in a 50 mL conical centrifuge tube, freeze the samples at −80°C.
    **CRITICAL:** To increase the efficiency of freeze-drying in the later step, the tubes should be tilted slightly while freezing so that the surface area of the frozen sample becomes larger (Figure 5A). However, it should be done in a stable place in a deep freezer.
    **CRITICAL:** Do not use tube stands with insulation materials such as Styrofoam during freezing (Figure 5B), as the temperature difference inside the tube will delay the timing of freezing at the bottom, resulting in tube breakage.
    **Pause point:** The sample has a sufficient sex-inducing activity for 6 months when stored at −80°C. Longer periods may be acceptable but have not been tested.

### Freeze-drying of the extracted supernatants (Day 2–5)

**Timing: 4 days**

Freeze-dry the extracted supernatant. Follow the instructions for the use of the available lyophilizer.

8. Preparation for freeze-drying.
  a. Turn on a lyophilizer and set it to low temperature and low pressure.
  b. Chill the lyophilization bottle (the part of the glass bottle in Figure 5C) at −20°C.
  c. Take off the lid of the 50 mL tubes containing the sample.
  d. Cover it with a sheet of medicinal powder paper.
  e. Secure it with a rubber band, and puncture about 10 holes with a needle (e.g., TERUMO syringe needle 18G, TERUMO, Japan) (Figure 5D).
  f. Keep it at −80°C until set in the lyophilizer.

***Note:*** The rubber part (Figure 5C) of the lyophilization bottle will harden when cooled, making it difficult to fit, so cool the glass part (Figure 5C) only.

***Note:*** Any freeze-dried sample powder that may get on the medicinal powder paper lid can be flicked off with a finger and dropped into the tube.

9. Perform freeze-drying.
  a. Place the sample tube upright in the lyophilization bottle and quickly place it in the lyophilizer (Figure 5C).
    ***Note:*** Wear gloves (1) to prevent the sample from melting at your body temperature and (2) to protect your hands since the bottles are very cold, but be aware that the tubes and bottles become slippery.
  b. Immediately open the cock (from the state “ventilation” shown in Figure 5E to the state “vacuum” shown in Figure 5F) and start freeze-drying.
    **CRITICAL:** Do this quickly before the sample thaws. If it thaws even slightly, freeze again. However, the sample freshly removed from −80°C will not thaw immediately, so set it calmly.
    **CRITICAL:** Be aware of the sunlight and UV light throughout the entire protocol, since the sex-inducing substance is an unknown compound, and the possibility of degradation by UV cannot be ruled out. Use aluminum foil as appropriate (e.g., Figure 5G).
  c. Allow 3–4 days to complete freeze-drying.
    **Pause point:** The freeze-dried powder can be stored at −80°C with the tubes upright. The sample has sufficient sex-inducing activity for 6 months when stored at −80°C. Longer periods may be acceptable but have not been tested.

### Fractionation by open-column chromatography (Day 6–7)

**Timing: 2 days**

In this step, the extracted supernatant is fractionated by open-column chromatography to Fr. M0, M10, M30, M50, and M100 (Figure 3). The sex-inducing substance is eluted in Fr. M30.

The reason for fractionating into five fractions is as follows. Since the sex-inducing substance is an unknown compound, it cannot be ruled out that the elution time may shift due to, for example, the differences in the pH of the extract, which might vary depending on the type of flatworm used (e.g., species difference and dietary conditions). Therefore, Frs. M10 and M50, which elute before and after the elution time of Fr. M30, should also be prepared just in case Fr. M30 does not have sex-inducing activity. Extremely hydrophilic or hydrophobic substances elute in Frs. M0 and M100 and will be removed from the analysis.

Unless specified, all steps should be performed at 4°C or on ice.

***Note:*** The time required for chromatography will vary depending on the type and batch of flatworms used. Including preparation, it takes an average of 7–8 h for the terrestrial planarian *B. nobile* and 10–11 h for the fluke *C. calicophorum*.

10. Prepare and wash ODS gel (column packing material) at 20°C–23°C.
  ***Note:*** We recommend washing ODS gels the day before chromatography.
  **CRITICAL:** MeOH should be handled in a fume hood, and appropriate safety gloves and goggles should be worn. Collect and dispose the waste liquid appropriately.
  a. Weigh 50 g of the ODS gel, Cosmosil 75C18-OPN (Nacalai Tesque, Japan), and place it in a 500 mL beaker.
  b. Add at least three column volumes (i.e., 300 mL in this case) of 100% MeOH.
    ***Note:*** HPLC grade MeOH is not required ONLY for step 10. For example, EP (Extra Pure Reagent) grade MeOH (Nacalai Tesque, Japan) can be used.
  c. Wash the ODS gel for 30 min, stirring once every 5 min, for example, with a clean spatula.
  d. Allow to stand for 5 min.
    **CRITICAL:** During the 5 min, the ODS gel floating in MeOH will fall to the bottom of the beaker.
  e. Slowly discard the MeOH for about 1 min to prevent the ODS gel from floating and flowing away.
  f. Repeat steps b–e two more times for a total of three washes with 100% MeOH.
  g. After the final wash, add 300 mL of 100% MeOH and store it at 20°C–23°C.
    ***Note:*** When adding new 100% MeOH, rinse off any ODS gel adhering to the beaker wall with a glass pipette after discarding the MeOH.
    ***Note:*** Be careful not to let the ODS gel dry out.
11. Prepare the following MeOH solutions to be used in step 17 and keep them on ice.

100% MeOH:300 mL.

50% aqueous MeOH:300 mL.

Milli-Q water:300 mL.

12. Prepare six different containers for fraction collection in step 18 (e.g., wide-mouth 500 mL PP bottle Eyeboy, AS ONE, Japan).

**Table:** Preparation of bottles

Labeling	Liquid volume to be collected in the bottle (mL)
“Flow-through”	160
“Fr. M0”	240
“Fr. M10”	300
“Fr. M30”	300
“Fr. M50”	300
“Fr. M100”	300

**CRITICAL:** Use bottles that will not break at −80°C.

***Note:*** It may be a good idea to draw a line at the bottle for each liquid volume.

13. Prepare three column volumes (i.e., 300 mL) of 0%, 10%, 30%, 50%, and 100% MeOH solutions to be used in step 18 as follows and keep them on ice.

**Table:** Preparation of solution

Solution	MeOH to add (mL)	Milli-Q water to add (mL)	Total volume (mL)
0% MeOH (M0)	0	300	300
10% aqueous MeOH (M10)	30	270	300
30% aqueous MeOH (M30)	90	210	300
50% aqueous MeOH (M50)	150	150	300
100% MeOH (M100)	300	0	300

***Note:*** Prepare them on the same day because MeOH volatilization changes the concentration.

**Figure 6:**
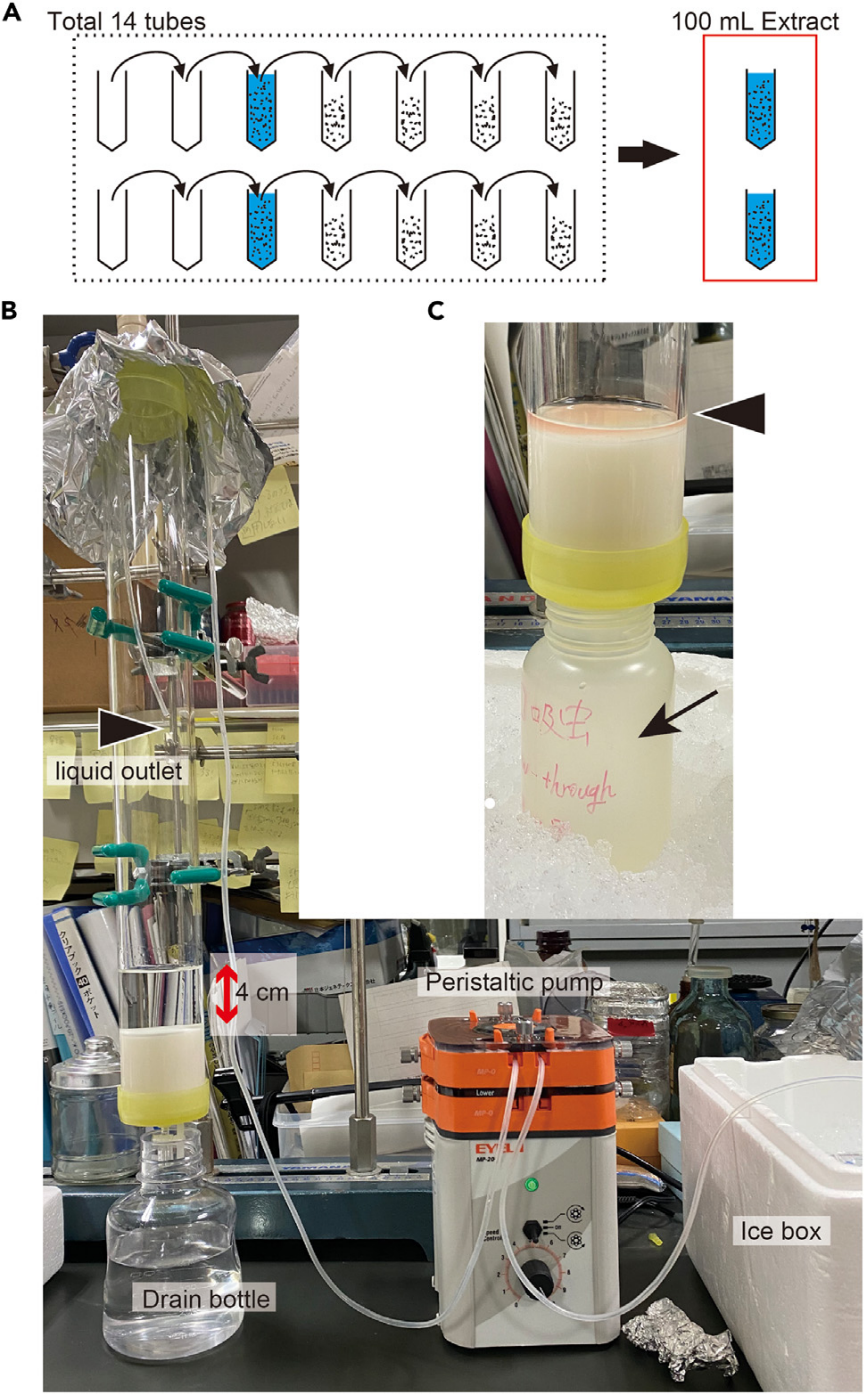
Open-column chromatography (A) Schematic diagram showing how to dissolve the sample powder in 100 ml of Milli-Q water. (B) Open-column chromatography system. (C) View of the column and collection bottle (arrow) just before adding the next solution. Wait for the previous solution to drop just above the ODS gel (arrowhead) before adding the next solution.
    iii. Repeat this process and finally centrifuge all the tubes shortly to collect all the samples, obtaining 100 mL of sample solution for the two 50 mL conical centrifuge tubes.
      ***Note:*** Do not refreeze the sample solution once it is dissolved.
  c. Filtrate the sample solution in multiple small volumes using a 10 mL syringe (e.g., TERUMO Disposable 10 mL syringe, TERUMO, Japan) and a 0.45 μm filter (e.g., Millex-HV Syringe-driven 0.45 μm PVDF Filter Unit, Merck Millipore, USA).
    ***Note:*** Filtration must be performed because in the process of freezing, drying, and dissolving in water, micelles in the ultracentrifuged supernatant may appear as small insoluble particles.
    ***Note:*** Perform filtration slowly in small portions. Applying too much pressure when the filter becomes clogged would result in the filter coming off vigorously and scattering of the sample.
    ***Optional:*** Filtration requires a lot of force and filters, so if it seems impossible, Rapid-Flow 0.2 μm PES Filters (Nalge Nunc International, USA) can be used. However, using Rapid-Flow filters are not recommended because it will cause additional sample loss.
    ***Note:*** When the filter becomes clogged, replace it with a new one. To reduce sample loss, point the mouth of the syringe upward before removing the filter, pull the plunger back slightly to let air in, and collect the remaining sample in the filter into the syringe.
  d. Keep the samples on ice until step 18.
15. Set up an open column Econo-Column (50 φ × 500 mm) (Bio-Rad, USA) and a peristaltic pump (Figure 6B).

***Note:*** The liquid outlet should be positioned above the ODS gel (Figure 6B, arrowhead), because the momentum of the liquid flow delivered by the peristaltic pump may gouge the ODS gel layer.

14. Prepare the sample solution for chromatography (the flatworm extract).
  a. Keep the freeze-dried supernatant powder in 14 tubes on ice.
  b. Dissolve the powder in 100 mL of Milli-Q water.
    i. Add 50 mL of Milli-Q water to the first and second tubes containing the powder and dissolve the solution by gently pipetting it on ice using a long Pasteur pipette.
    ii. Transfer the solution in each tube to the other two tubes containing the powder using a long Pasteur pipette and dissolve the sample in the same manner on ice (Figure 6A).

16. Pack the ODS gel to the column (Figure 6B).
  a. Open the column faucet.
  b. Pack the ODS gel to the column and collect the drained MeOH as appropriate (Figure 6B).
  c. Collect and pack any remaining ODS gel adhering to the beaker and the wall of the column by rinsing with MeOH.
17. Replace MeOH in the ODS gel with Milli-Q water and prepare for chromatography. Use the solution prepared in step 11.
  a. Discard the original MeOH.
  b. Flow 300 mL of 100% MeOH using a peristaltic pump (flow rate: 10 mL/min).
  c. Flow 300 mL of 50% aqueous MeOH using a peristaltic pump (flow rate: 10 mL/min).
  d. Flow 300 mL of Milli-Q water using a peristaltic pump (flow rate: 10 mL/min).

**CRITICAL:** Wait for the previous solution to drop just above the ODS gel (Figure 6C, arrowhead) before adding the next solution.

**CRITICAL:** Do not expose the ODS gel to air.

***Note:*** The flow rate is set at 10 mL/min, but if the ODS gel is gouged, it should be slowed down accordingly. Moreover, it may take some time for the solution to pass through the column. When using a column as the one used in this study (5 cm i.d.), make sure that the volume of the solution does not exceed a height of 4 cm above the ODS gel (Figure 6B, indicated in red line) by adjusting the flow rate of the peristaltic pump accordingly. This keeps the flow rate at the optimum level for reproducible fractionation.

***Note:*** All solutions passing through the peristaltic pump should be ice-cooled (Figure 6B, white arrow). It would be ideal to perform the chromatographic work in a cold room when purifying an unknown compound such as the sex-inducing substance, because the sample still contains various components such as digestive enzymes. However, since this was not possible for us due to space limitations, we opted the protocol described above and were able to recover the sex-inducing substance.

***Note:*** Room temperature should be kept constant at 20°C.

18. Perform chromatography to produce Frs. M0-M100.
  a. Elution of flow-through and Fr. M0.
    i. When the water in step 17-d flows through and the liquid level is just above the ODS gel, start flowing 100 mL of the sample solution (flatworm extract) with a peristaltic pump.
    ii. Simultaneously with step (i), place the bottle labeled "Flow-through" under the column with ice (Figure 6C, arrow) and collect the eluate.
    iii. When the liquid level is just above the ODS gel, start flowing 300 mL of 0% MeOH (M0) with a peristaltic pump.
      ***Note:*** The volume of liquid that the ODS gel in the column can hold is approximately 60 mL. Thus, the "flow-through" here is defined as the 160 mL eluate, consisting of 60 mL of the water initially contained in the column before the sample flowed through and 100 mL of the sample. The end of the "flow-through" collection is marked when 60 mL of liquid elutes after the 0% MeOH solution is begun to be added.
    iv. When the eluate has accumulated to the 160 mL line of the "flow-through" bottle (i.e., when 60 mL has eluted since 0% MeOH began flowing), switch to the collection bottle labeled "Fr. M0".
    v. Collect the eluate until the liquid level is just above the ODS gel (i.e., 240 mL of Fr. M0 in the bottle).
      ***Note:*** From the next step, Fr. M10 is defined as the liquid eluted from the point when 10% aqueous MeOH (M10) begins to flow. Thereafter, Fr. M30 to M100 are defined in the same manner.
  b. Elution of Fr. M10 (i.e., the fraction eluted from when 10% aqueous MeOH began to flow).
    i. When the liquid level from the previous step is just above the ODS gel, start flowing 300 mL of 10% aqueous MeOH (M10) with a peristaltic pump.
    ii. Simultaneously with step (i), switch to the collection bottle labeled "Fr. M10".
    iii. Collect the eluate until the liquid level is above the ODS gel (i.e., 300 mL of Fr. M10 in the bottle).
  c. Elution of Fr. M30 (i.e., the fraction eluted from when 30% aqueous MeOH began to flow).
    i. When the liquid level from the previous step is just above the ODS gel, start flowing 300 mL of 30% aqueous MeOH (M30) with a peristaltic pump.
    ii. Simultaneously with step (i), switch to the collection bottle labeled "Fr. M30".
    iii. Collect the eluate until the liquid level is above the ODS gel (i.e., 300 mL of Fr. M30 in the bottle).
  d. Elution of Fr. M50 (i.e., the fraction eluted from when 50% aqueous MeOH began to flow).
    i. When the liquid level from the previous step is just above the ODS gel, start flowing 300 mL of 50% aqueous MeOH (M50) with a peristaltic pump.
    ii. Simultaneously with step (i), switch to the collection bottle labeled "Fr. M50".
    iii. Collect the eluate until the liquid level is just above the ODS gel (i.e., 300 mL of Fr. M50 in the bottle).
  e. Elution of Fr. M100 (i.e., the fraction eluted from when 100% MeOH began to flow).
    i. When the liquid level from the previous step is just above the ODS gel, start flowing 300 mL of 100% MeOH (M100) with a peristaltic pump.
    ii. Simultaneously with step (i), switch to the collection bottle labeled "Fr. M100".
    iii. Collect the eluate until the liquid level is above the ODS gel (i.e., 300 mL of Fr. M100 in the bottle).
  f. Finally, close the faucet of the column.
    ***Note:*** The obtained fractions should be stored at 4°C, if the next step (sample concentration) is to be performed the next day, otherwise at −80°C.
    ***Note:*** ODS gels can be reused after washing as described in step 10 and should be stored in 100% MeOH. However, reuse is not recommended if the gel is heavily contaminated with mucus or dye. If reuse is still necessary, wash three times with 100% chloroform (increase the waiting time from 5 min to about 7.5 min because the gels do not sink easily to the bottom of the beaker in chloroform), three times with 100% EtOH, and then three times with 100% MeOH. Nevertheless, limiting the reuse to 2–3 times would be best.
    **CRITICAL:** Chloroform should be handled in a fume hood, and appropriate safety gloves and goggles should be worn. Collect and dispose the waste liquid appropriately.
    **Pause point:** The sample has sufficient sex-inducing activity for 6 months when stored at −80°C. Longer periods may be acceptable but have not been tested.

### Concentration of Fr. M30 using a rotary evaporator (Day 8)

**Timing: 1 day**

Fr. M30 is concentrated from 300 mL to 1.6 mL using a rotary evaporator because it contains an organic solvent (MeOH). The method is the same for other fractions. The higher the water content, the longer it takes to concentrate. We describe our system here, but follow the instructions for the use of the available machine.

**CRITICAL:** Samples containing MeOH should be handled in a fume hood or well-ventilated area, and appropriate safety gloves and goggles should be worn. Collect and dispose the MeOH waste liquid appropriately.

Unless specified, all steps should be performed at 20°C–23°C.

**Figure 7:**
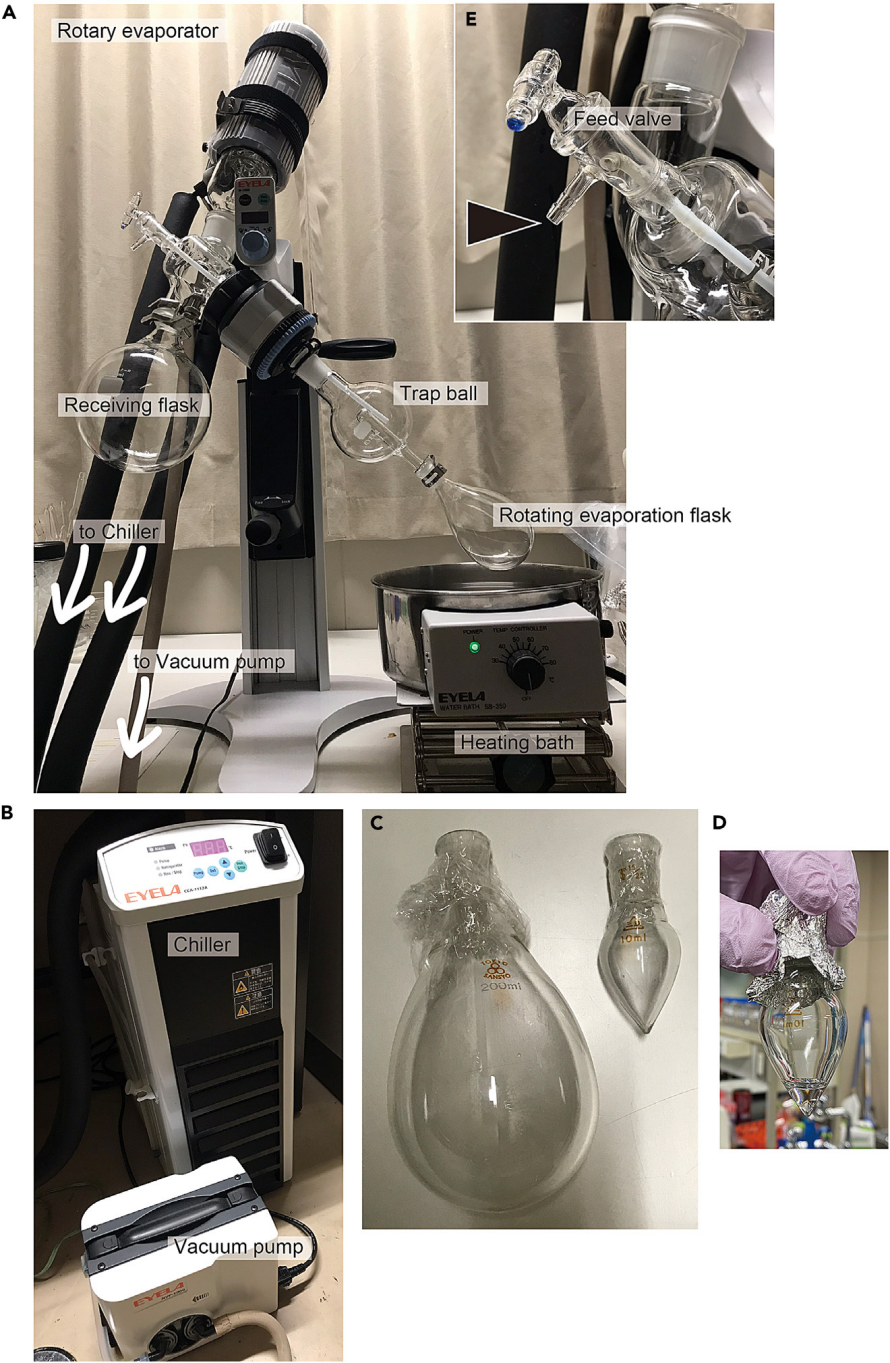
Concentration by a rotary evaporator (A) Rotary evaporator system. (B) Vacuum pump and chiller. (C) Eggplant-shaped evaporation flask (left) and pear-shaped evaporation flask (right). (D) Sample concentrated to approximately 800 μL in a pear-shaped flask. (E) Feed valve.
  b. Switch on the vacuum pump (Figure 7B).
  c. Switch on the cooling chiller at −14°C (Figure 7B; using 50% ethylene glycol as the refrigerant).
  d. Set a water bath at 37°C (Figure 7A).
21. Add approximately 60 mL of Fr. M30 into a 200 mL eggplant-shaped evaporation flask (Figure 7C, left) with a long Pasteur pipette.
22. Attach the flask to the evaporator and start the rotations, carefully observing it in case of sudden boiling.

***Note:*** When the sample boils, stabilize the liquid surface by opening the air exhaust to loosen the decompression (90 rpm).

***Note:*** By opening the feed valve (Figure 7E) and applying pressure on the hole (Figure 7E, arrowhead) using a finger, one can promptly release the decompression by lifting the finger if required.

19. If the sample is frozen, thaw and keep it on ice until use (step 21).
20. Prepare for rotary evaporating. We describe our system but follow the instructions for the use of the available machine.
  a. Set the necessary parts for rotary evaporating (Figure 7A), except an evaporation flask.

23. When the liquid level has stabilized, slowly submerge the rotating evaporation flask into the water bath, carefully observing it in case of sudden boiling.

***Note:*** The indication that the liquid level has stabilized is when bubbles no longer emerge from the sample in the rotation flask and the liquid begins to drip into the receiving flask.

***Note:*** When the sample boils, pull the flask up and remove it from the water bath.

24. Concentrate until the sample is about 10 mL.

***Note:*** Never let it dry up.

25. Stop rotating and slowly open the air exhaust, carefully observing it in case of sudden boiling. When the liquid surface has stabilized, remove the evaporation flask.
26. Repeat steps 21–25 several times until the entire sample is placed in the evaporation flask and the sample bottle is empty.

***Note:*** Finally, when the sample bottle is empty, rinse it with a volume equal to 1/10th of the bottle's capacity (e.g., 50 mL for a 500 mL bottle) of Milli-Q water to collect any remaining sample. This collected rinse water should also be placed in the evaporation flask and concentrated.

27. After concentrating the sample (including the final rinse water) to about 5–6 mL, move the entire sample in the evaporation flask into a 50 mL conical centrifuge tube with the long Pasteur pipette.
28. Add about 2 mL of Milli-Q water, rinse the evaporation flask for 90 s while applying sonication evenly to the flask (e.g., using Ultrasonic cleaner USC-100Z38S-22, IWAKI, Japan) (Methods video S4), and collect as much of the remaining sample inside as possible.
29. Repeat step 28.

***Note:*** About 10 mL (5–6 + 2 + 2 mL) of concentrated sample is obtained.

30. Add 3 mL of the concentrate into a 10 mL pear-shaped evaporation flask (Figure 7C, right) with the long Pasteur pipette.
31. Concentrate in the same manner as described in steps 22–26 until the entire sample is moved to the evaporation flask and the sample tube is empty.

***Note:*** Finally, when the sample tube is empty, rinse it with a volume equal to 1/10th of the tube's capacity (e.g., 5 mL for a 50 mL conical centrifuge tube) of Milli-Q water to collect any remaining sample in the tube. This collected rinse water should also be placed in the evaporation flask and concentrated.

32. After concentrating the sample (including the rinse water) to approximately a final 800 μL (Figure 7D), move the entire sample from the evaporation flask into a 2 mL microcentrifuge tube using a P1000 micropipette set to 800 μL.

***Note:*** If the volume exceeds 800 μL, continue concentrating; if less, add Milli-Q water to bring the volume to 800 μL.

33. Add 400 μL of Milli-Q water with a P1000 micropipette, rinse the evaporation flask for 90 s while applying sonication evenly to the flask (e.g., using Ultrasonic cleaner USC-100Z38S-22, IWAKI, Japan) (Methods video S4) as in step 28, and collect as much of the remaining sample inside as possible.
34. Repeat step 33.

***Note:*** About 1.6 mL (800 + 400 + 400 μL) of concentrated sample is obtained.

35. Store at −80°C.

**Pause point:** The sample has sufficient sex-inducing activity for 6 months when stored at −80°C. Longer periods may be acceptable but have not been tested.

***Note:*** After concentration is finished, remove any organic solvent (i.e., MeOH in the sample) remaining in the vacuum pump, leaving the pump running for a while.

Methods video S4. How to rinse the evaporation flask while applying sonication, related to steps 28 and 33

### Fractionation of Fr. M30 by reverse-phase HPLC (Day 9)

**Timing: 1 day**

**Figure 8:**
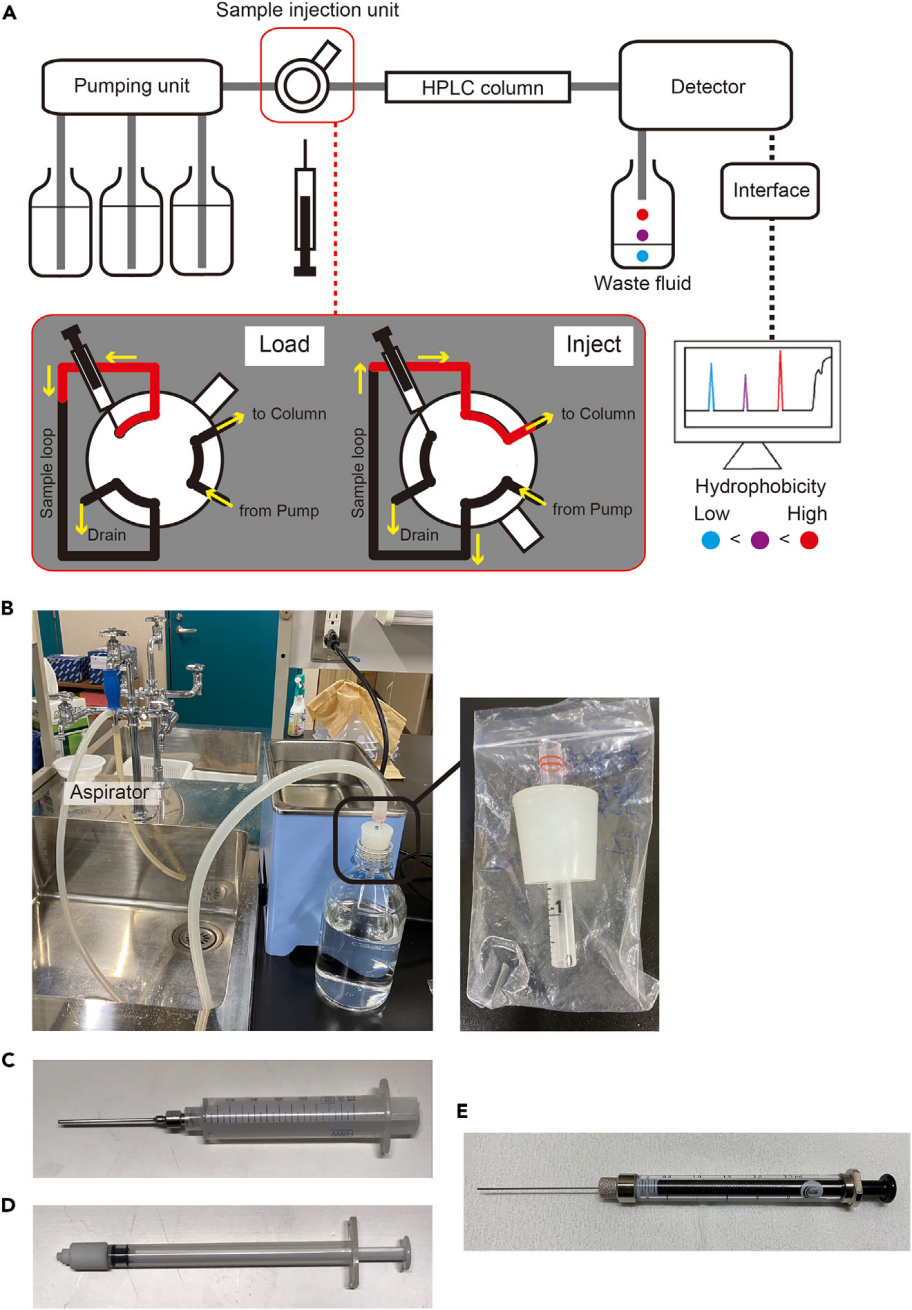
Principles of HPLC and some equipment The sample syringe (Figure 8E) is in direct contact with the sample, so it should be made of glass to avoid the risk of plastic-derived components mixing with the sample. Other syringes (Figures 8C and 8D) do not have this concern and may be made of plastic (less expensive than glass). (A) Schematic diagram of the HPLC system. (B) Solvent degassing system. (C) Luer-lock syringe. (D) Cleaning syringe. (E) Sample syringe.

The sex-inducing substance has already been purified to some extent with Fr. M30, but it can be further fractionated using reverse-phase HPLC. We describe our system here, along with a brief principle of HPLC (Figure 8A), but follow the instructions for the use of the available HPLC system.

The dissolved sample is injected into the preparative HPLC column with solvent (e.g., MeCN) called the mobile phase and moves through the column while interacting with the stationary phase (e.g., C30-UG-5, 20 φ × 250 mm). If the interaction with the mobile phase is strong, the sample component moves faster through the column in the flow of the mobile phase. On the other hand, if the interaction with the stationary phase is strong, the component moves slower. This difference in migration speed results in the separation of sample components, which is sequentially detected by the UV detector (290 nm) at the exit.

The sex-inducing substance is eluted immediately after the tryptophan retention time. Therefore, this protocol requires that the retention time of tryptophan be recorded prior to sample injection and the fractions containing sex-inducing substances be collected accordingly.

HPLC must be performed in a temperature-stable environment (e.g., using a column oven or a small, air-conditioned temperature-controlled room at 20°C) for better reproducibility, since the interaction between sample components and mobile and stationary phases is strongly affected by temperature.

Unless specified, all steps should be performed at 20°C.

36. Configure the settings on the available system accordingly, prior to the experiment. We used the ChromNav version 2 software.
  ***Note:*** This is the control method to be used in step 47-b-v.
  a. The conditions of the reversed-phase HPLC to be used in this study are as follows.
    i. UV detect: 290 nm.
    ii. Pumping system.

**Table:** Setting

Flow rate	5.0 [mL/min]
Upper pressure limit	25.0 [Mpa]
Lower pressure limit	0.0 [Mpa]
Solvent A	10% aqueous MeCN
Solvent B	100% MeCN
Solvent C	100% MeOH
Solvent D	NA
    iii. Time program (analysis time: 75 min).

**Table:** Setting

Time	Solvent ratio			
	A	B	C	D
INITIAL	95.0 [%]	5.0 [%]	0.0 [%]	0.0 [%]
18.00 [min]	90.0 [%]	10.0 [%]	0.0 [%]	0.0 [%]
53.00 [min]	70.0 [%]	30.0 [%]	0.0 [%]	0.0 [%]
55.00 [min]	60.0 [%]	40.0 [%]	0.0 [%]	0.0 [%]
55.10 [min]	0.0 [%]	0.0 [%]	100.0 [%]	0.0 [%]
37. Prepare a tryptophan solution to be used as an indicator for collecting fractions; add 20 mg tryptophan to 15 mL Milli-Q water (final 1.33 mg/mL).
38. Preparation of solvent for the mobile phase.
  a. Calculate the number of times you will run the program and the volume of the solution to be prepared, referring to the following table. The present protocol uses 4P+3E + C+100 (mL) of solution.

**Table:** Calculation of the amount of solution required

	The amount of solution required (mL)		
	10% aqueous MeCN	100% MeCN	100% MeOH
P: Running 1 program	229.75	45.25	100
E: Equilibration	213.75	11.25	0
C: Column storage (step 55-d)	124.875	250.125	0
100 mL as extra	100	100	100
4P+3E + C+100	1785.125	564.875	500
  b. Prepare the required amount of solution in a 2-L glass bottle (i.e., approximately 1800 mL of 10% aqueous MeCN solution, 570 mL of 100% MeCN, and 500 mL of 100% MeOH).
    ***Note:*** It is recommended to do this the day before the experiment.
39. Degas the solvent. Using a dedicated machine is fine. For example, we used the following system.
  a. Connect the bottles containing the solvent (prepared in step 38) to the aspirator (Figure 8B).
  b. Start depressurization and sonicate (e.g., using Ultrasonic cleaner USC-100Z38S-22, IWAKI, Japan) for 1–1.5 min (no more than 2 min to prevent volatilization of MeCN) while rotating the bottle (Methods video S5).
  c. When degassing is complete, remove the aspirator.

***Note:*** Slowly remove the rubber stopper of the aspirator from the tube, as the solvent now easily absorbs the gas.

***Note:*** For aspirators using a water supply, remove the aspirator and then shut off the water supply to prevent backflow.

**CRITICAL:** In HPLC, high pressure between the pump and the end of the column is suddenly released, which results in the formation of bubbles by the dissolved gases after the column. Since the bubbles result in detection noise, degassing should be done thoroughly.

***Note:*** Be careful not to splash solvent on the rubber stopper when rotating the bottle.

**Figure 9:**
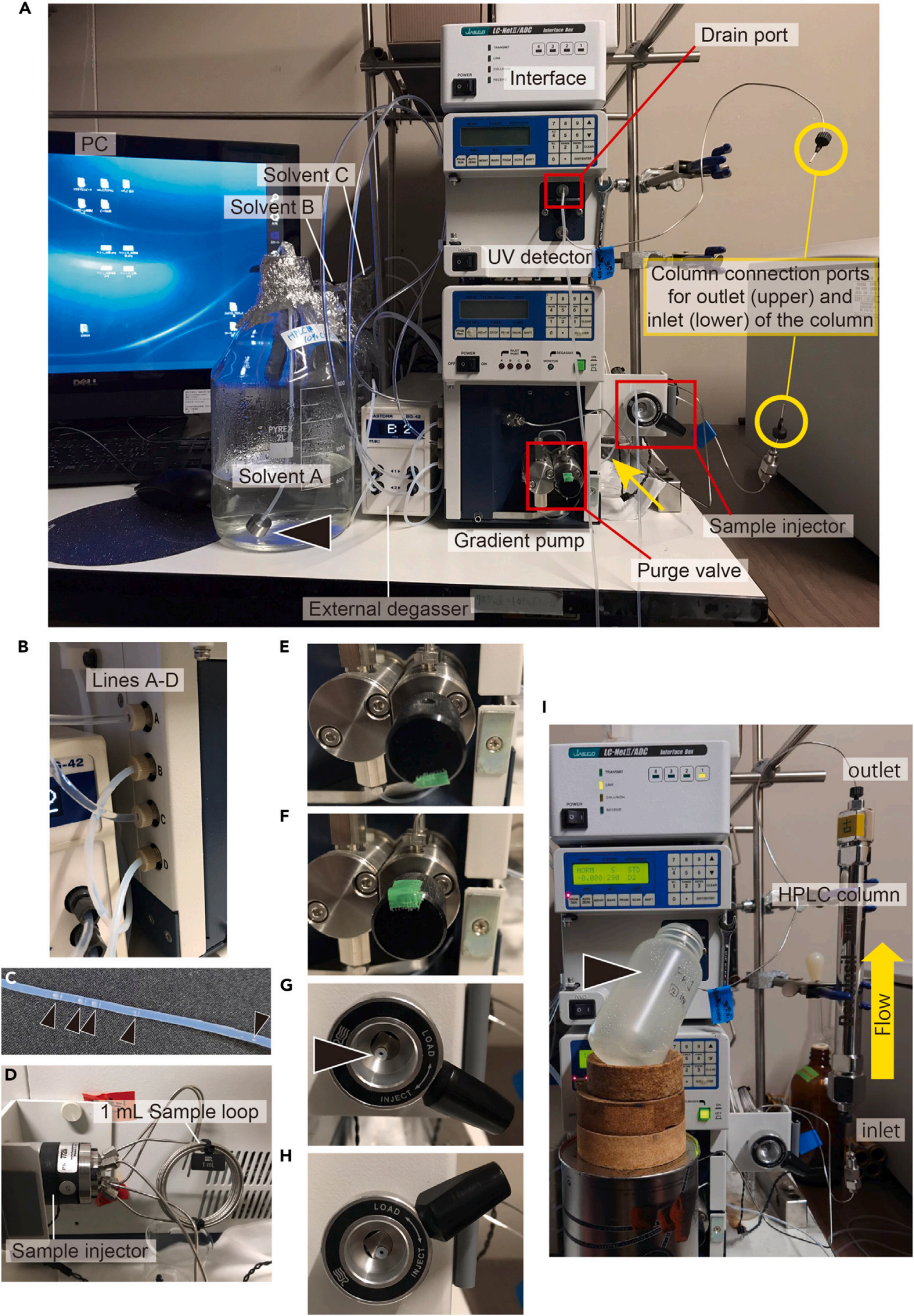
HPLC system (A) Image showing the entire HPLC system. (B) Lines A-D of the HPLC system. (C) Example of air bubbles in the tubing. (D) Sample loop and sample injector. (E) With purge valve turned. (I) HPLC column and collection bottle set up in the HPLC system. (F) With purge valve turned back. (G) Sample injector knob in the down position. (H) Sample injector knob in the upper position.
41. Attach a sample loop for 1 mL (Figure 9D), since the maximum volume of liquid injected in this protocol is 800 μL.

***Note:*** The sample loop must be of appropriate size to minimize the sample lost during injection.

***Note:*** Dip the end of the tubing firmly into the solvent in the bottle (Figure 9A, arrowhead). Cover the mouth of the bottle with plastic wrap and aluminum foil to prevent dust (Figure 9A).

40. Set the bottle containing 10% aqueous MeCN in line A, the bottle containing 100% MeCN in line B, and the bottle containing 100% MeOH in line C of the HPLC system (Figures 9A and 9B).

42. Priming the HPLC pump (i.e., filling the inside of the device with the mobile phase).
  a. Switch on the gradient pump (Figure 9A).
  b. Switch on the degasser (Figure 9A).
    ***Note:*** We are using an external degasser. The manufacturer's instructions state to always leave them on, even when not using the HPLC.
    **CRITICAL:** The main flow rate used in the protocol is 5 mL/min. Check the flow rate limits at which the internal degasser of the available gradient pump can properly handle air. Use an external degasser if necessary, as the pump's internal degasser will break down if used for a long time beyond its capacity.
  c. Priming from the line A to the purge valve outlet.
    i. Set the gradient pump to flow solvent A (i.e., set the solvent ratio A: 100%, B: 0%, C: 0%, and D: 0% on the pump) at a flow rate of 10 mL/min.
    ii. Connect a Luer-lock syringe (Figure 8C) to the drainage tubing (Figure 9A, yellow arrow).
    iii. Turn the purge valve as shown in Figure 9E, which connects the line to the drainage.
    iv. Press the "PRGM RUN" button on the pump to make it start.
    v. While pulling the plunger of the Luer-lock syringe, let solvent A flow until the syringe is full (i.e., 10 mL).
    vi. Press the "PUMP" button to make it stop.
    vii. Turn the purge valve back as shown in Figure 9F, which closes the connection to the drainage.
    viii. Remove the syringe from the drainage tubing, and discard the liquid waste inside.
    ix. Repeat steps ii-viii several times until no air bubbles (Figure 9C, arrowhead) are seen in, for example, the tubing.
      **CRITICAL:** Confirm that no air bubbles are seen. In our case, bubbles are usually no longer visible after twice the procedure.
  d. Priming from line B to the purge valve outlet.
    i. Set the gradient pump to flow solvent B (i.e., set the solvent ratio A: 0%, B: 100%, C: 0%, and D: 0% on the pump) at a flow rate of 10 mL/min.
    ii. Repeat the previous step c-ii to c-ix several times until no air bubbles are seen in the tubing.
  e. Priming from the line C to the purge valve outlet.
    i. Set the gradient pump to flow solvent C (i.e., set the solvent ratio A: 0%, B: 0%, C: 100%, and D: 0% on the pump) at a flow rate of 10 mL/min.
    ii. Repeat the previous step c-ii to c-ix several times until no air bubbles are seen in the tubing.
  f. Priming from the line between the purge valve outlet and the column connection port.
    i. Keep the purge valve in a state as shown in Figure 9F, which closes the connection to the drainage.
    ii. Set the gradient pump to flow 15% aqueous MeCN, which is the same condition as the INITIAL state of the program set in step 36-a-iii (i.e., set the solvent ratio A: 95%, B: 5%, C: 0%, and D: 0% on the pump) at a flow rate of 10 mL/min.
    iii. Press the "PRGM RUN" button on the pump to make it start.
    iv. Let the solvent flow until approximately 20 mL of liquid comes out of the column connection port.
      ***Note:*** Collect the waste liquid appropriately, for example, by attaching a tube to the connection port.
    v. Press the "PUMP" button to make it stop.
      **CRITICAL:** Ensure no air bubbles are present in the liquid that fills the system.
43. Set the preparative HPLC column Develosil C30-UG-5 (20 φ × 250 mm) (Nomura Chemical, Japan).
  a. Check the orientation of the flow (Figure 9I) and set it on the stand.
  b. Set the gradient pump flow rate to 1 mL/min (the solvent ratio is the same as in step 42-f-ii).
  c. Press the "PRGM RUN" button on the pump to make it start.
  d. With the solvent flowing, remove the end cap at the inlet of the column, press the column connection port against it, and tighten the connector properly.
  e. After confirming the increase in pressure, quickly remove the column outlet end cap, confirm that the liquid has exited, and tighten the connector properly in the same manner while pressing the other column connection port against it.
    ***Note:*** Avoid air bubbles.
    ***Note:*** If the connection port is not firmly pressed against the column, the dead volume will occur, resulting in uneven pressure on the column.
    ***Note:*** Tighten the connectors properly and ensure no liquid leaks during the experiment.
  f. After confirming that the liquid comes out of the drain port of the UV detector (Figure 9A), press the "PUMP" button on the pump to make it stop.
44. Cleaning the sample injector and the sample syringe.
  a. Cleaning the sample injector (Figure 9A) as shown in Methods video S6.
    i. Make sure the sample injector knob is in the down position (Figure 9G).
    ii. Fill the cleaning syringe (Figure 8D) with the following fluid and push its outlet into the center of the injector knob (Figure 9G, arrowhead).
    iii. Inject 3 mL MeOH by pushing the plunger and remove the syringe.
    iv. Inject 3 mL Milli-Q by pushing the plunger and remove the syringe.
  b. Cleaning the sample syringe (Figure 8E).
    i. Suck and discard 100% MeOH, repeating three times.
    ii. Suck and discard Milli-Q water, repeating three times.
45. Sample filtration.
  a. Thaw the sample (concentrated Fr. M30) and keep it on ice.
  b. Fill a 1 mL syringe (e.g., TERUMO Disposable 1 mL syringe, TERUMO, Japan) with the sample using a P1000 micropipette and filtrate it using a 0.2 μm filter for HPLC (e.g., mdi PVDF 0.2 μm Syringe Filter, Advanced Microdevices, India) into 2 mL microcentrifuge tube.
  c. Keep the sample on ice until use (step 52).

***Note:*** Filtration must be performed because in the process of freezing and thawing some components may precipitate as small insoluble particles.

***Note:*** Steps 44 and 45 can be done during the waiting time in steps 48 and 49 (wash & equilibration).

46. Switch on the UV detector and the interface in this order (these should be switched on before launching the ChromNAV ver. 2 software), and PC (Figure 9A).
47. Setting up the software for HPLC.
  a. Launch the software ChromNAV ver. 2.
  b. Configure the settings on the system referring to the following.
    i. Create the project (e.g., “Fractionation of the sex-inducing substance”).
    ii. Select “Manual measurement”.
    iii. Enter a sequence name (e.g., Date); a folder with this name will be created.
    iv. Enter a chromatogram name (e.g., “wash” for the first round; thereafter, “Tryptophan” and “M30”).
    v. Select the control method (created in step 36).
    vi. Time: 75.0 min.
    vii. Injection volume: 800 (μL).
48. Wash of the preparative HPLC column.
  a. Click "Start".
  b. Move the sample injector knob up (Figure 9H) and down (Figure 9G).
    ***Note:*** In our system, the "up and down" action of the sample injector knob sends a start signal to the software to initiate a gradient elution program. Without this action, the solution would continue to flow under the solvent ratio set as INITIAL.
  c. The 75-min gradient elution program set in step 36 is initiated (i.e., wash of the preparative HPLC column).
49. Equilibration of the preparative HPLC column.
  a. Click "Edit" to change the chromatogram name (e.g., from "wash" to “Tryptophan”).
  b. Click "Start".
  c. Let the program run for 45 min in the INITIAL status (i.e., equilibration).

***Note:*** Equilibration time is calculated based on the column volume. We flow three column volumes of the solvent. Since the flow rate of the preparative HPLC column (ø20 × 250 mm) is 5 mL/min, the time for three column volumes of the solution to flow is about 47 min.

***Note:*** When no peaks are detected and the line is flat, equilibration is complete, ready for the sample injection. This step can be done for more than 45 min until no peaks are detected.

**Figure 10:**
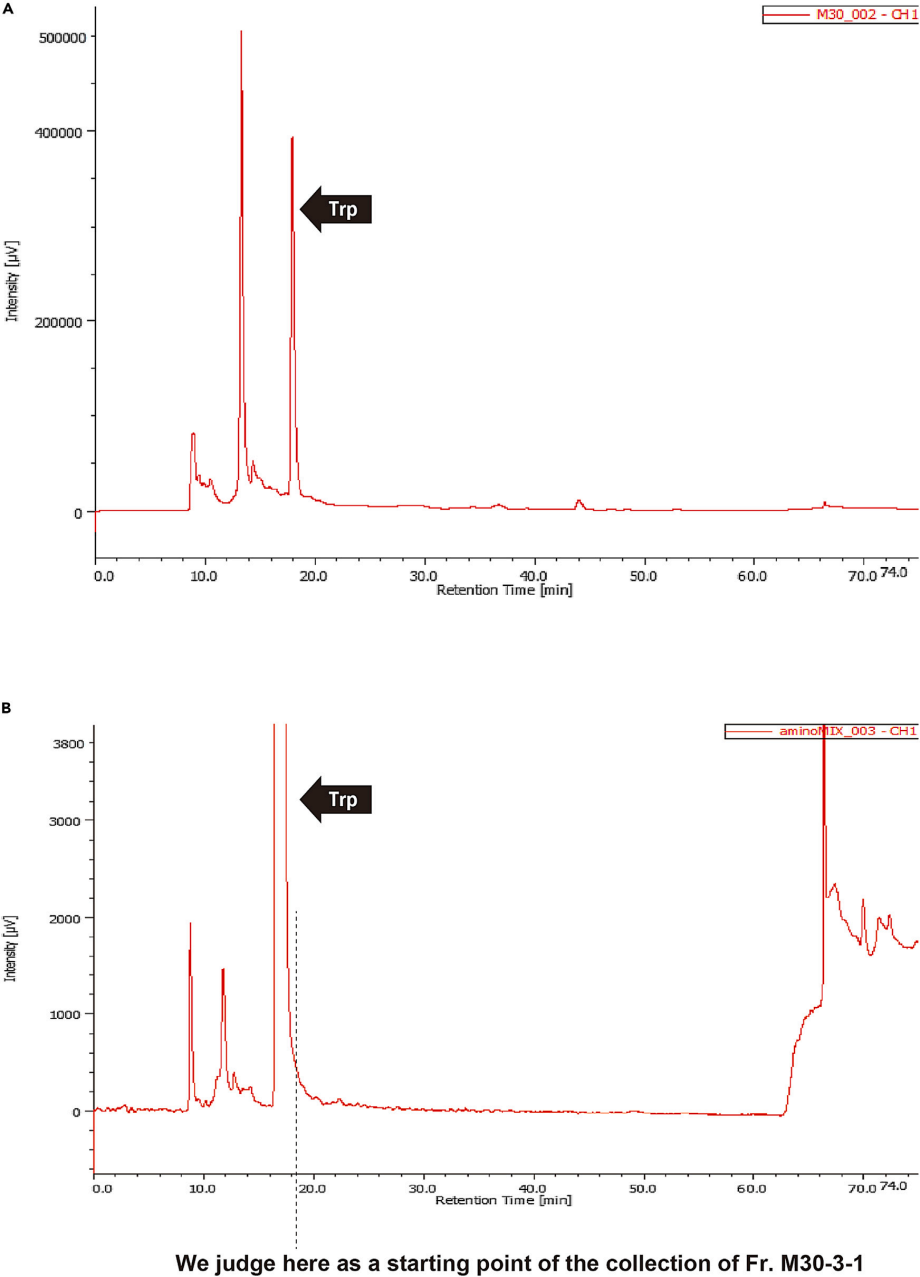
Example of determination of the base of tryptophan peak (A) Chromatogram showing the amount of tryptophan in Fr. M30 of *Calicophoron calicophorum*. (B) Example of peak-base judgment.
51. Cleaning the sample injector and syringe, as described in step 44.
52. Injection of Fr. M30 and collecting the fraction with sex-inducing activity.
  a. Click "Edit" to change the chromatogram name (e.g., "M30″).
  b. Click "Start".
  c. Let the program run for 45 min in the INITIAL status (i.e., equilibration).
  d. Fill the sample syringe with 800 μL of Fr. M30 by sucking and inject the sample as described in steps 50b and 50c.
    ***Note:*** Divide 1.6 mL of concentrated Fr. M30 in two fractions of 800 μL each. Injecting the entire volume at once will result in a broader peak of tryptophan in the sample, and more tryptophan will be introduced into the fraction collected from the reference point.
  e. Collect the active fraction.
    i. Magnify the scale of the intensity of the UV absorption chromatogram on the monitor to about Max intensity of 2,000.
    ii. As soon as reaching the reference time recorded in step 50d, set a collection bottle (e.g., wide-mouth 250 mL PP bottle Eyeboy, AS ONE, Japan) at the drain (Figure 9I, arrowhead).
    iii. Collect the fraction eluting for 6 min from the reference.
  f. After the 75-min program is completed, let the program run for another 45 min in the INITIAL state (i.e., equilibration).
  g. Repeat steps d–f for the other half of the 800 μL sample and collect the fraction.
  h. Finally, about 60 mL (30 mL twice) of the active fraction is obtained.
    ***Note:*** It is called Fr. M30-3-1 in a recent study by Sekii et al. (2023),^1^ and it is the fraction containing the most highly purified sex-inducing substance so far.
    ***Note:*** No consistently evident peaks have been identified in Fr. M30-3-1.
    **CRITICAL:** Since retention times may shift due to differences in column size (analytical or preparative) and experimental date, be sure to use a preparative column to flow tryptophan solution and check its retention time on the same day as the sample fractionation.
    **CRITICAL:** Changes in temperature will cause a shift in the HPLC retention time. Be sure to make the room temperature stable or use a column oven. Our room temperature is 20°C.
    **CRITICAL:** Match the timing of the peak detection on the monitor and the timing of the solution coming out of the drain port. It is recommended that the fractions of the tryptophan peak and the fractions eluting before and after the peak be collected beforehand using tryptophan solution and analyzed again by HPLC.
53. Click "Stop" after the 75-min program is completed.
54. Store the sample at −80°C.

***Note:*** An alternative approach would be to proceed directly to step 56 (i.e., removal of MeCN and concentration of the sample).

50. Injection of tryptophan solution and recording of its retention time.
  a. Fill the sample syringe (Figure 8E) with 100–150 μL of tryptophan solution by sucking.
    ***Note:*** Although the volume of injection is 100–150 μL, the same sample loop (1 mL) is used to keep the conditions consistent.
  b. To remove air bubbles, point the syringe outlet upward, tap gently to collect any air bubbles at the top, and push the plunger out slightly.
  c. Inject the sample. The operation in our HPLC system would be as follows (Methods video S7).
    i. Make sure the sample injector knob is in the down position and insert the sample syringe outlet into the center of the injector knob until it clicks.
    ii. Turn the sample injector knob up.
    iii. Push the plunger to load the sample.
    iv. Put the sample injector knob back down, which will send a start signal to the software and the sample will be injected into the column.
    v. The 75 min-program will begin.
    vi. Remove the syringe.
    Methods video S7. How to inject the sample into the HPLC column, related to step 50c
  d. Check and record the retention time at the base of the tryptophan peak, which will be the reference for starting to collect the fraction with sex-inducing activity.
    ***Note:*** An injection of 100–150 μL of tryptophan solution (1.33 mg/mL) yields an intensity of 300,000 to 400,000, which is about the same amount of tryptophan in the sample when the sex-inducing substance is obtained from Fr. M30 of *C. calicophorum* (Figure 10A). An example of peak-base judgment is shown in Figure 10B.
    ***Note:*** Since the width of the tryptophan peak base varies with the amount in the sample, a tryptophan solution of known concentration is used to set the reference.

55. HPLC termination. Be sure to follow the instruction manual of the available machine and perform the termination operation correctly.
  a. Exit ChromNAV ver. 2 and shut down the PC.
  b. Turn off the interface.
  c. Turn off the UV detector.
  d. Removal and storage of the preparative HPLC column.
    i. Set the gradient pump to flow 70% aqueous MeCN (i.e., set the solvent ratio A: 33.3%, B: 66.7%, C: 0%, and D: 0%) at a flow rate of 5 mL/min.
      ***Note:*** The manufacturer recommends storing columns in 70% aqueous MeCN solution.
      ***Note:*** This operation also serves to reset the pump settings (since the data of ChromNAV gradient elution program remains in the pump).
    ii. Press the "PRGM RUN" button on the pump and let the 70% aqueous MeCN solution flow for 75 min.
      ***Note:*** We flow 5 column volumes of solvent.
    iii. Change the pump setting to a flow rate of 1 mL/mL. Remove the tubing and cover the column outlet with an end cap; quickly do the same for the inlet (Figure 9I).
    iv. Store at 20°C–23°C.
  e. Turn off the gradient pump.

Methods video S5. How to degas the solvent, related to step 39

Methods video S6. How to wash the sample injector, related to step 44

### Concentration of Fr. M30-3-1 using a rotary evaporator (Day 10)

**Timing: 1 day**

The fractions obtained by reverse-phase HPLC contain MeCN, which must be removed for subsequent experiments. This is done using a rotary evaporator, which also serves the sample concentration.

**CRITICAL:** Samples containing MeCN should be handled in a fume hood or well-ventilated area, and appropriate safety gloves and goggles should be worn. Collect and dispose the MeCN waste liquid appropriately.

Unless specified, all steps should be performed at 20°C–23°C.

56. Concentrate the Fr. M30-3-1 from 60 mL (i.e., 30 mL collected twice) to 2–3 mL as described in steps 19–34.

***Note:*** The starting material comprises of 8 g (wet weight) of flatworms. When concentrated to a volume of 2.4 mL, a 300 μL quantity yields the active fraction that originates from 1 g (wet weight) of flatworms. This makes the calculation easier to perform.

57. Store at −80°C. If a feeding bioassay is performed to confirm the activity, mix with BSA before freezing (see step 58 for details) and lyophilize.

### Confirmation of sex-inducing activity of the obtained fraction by feeding bioassay (Day 11–41)

**Timing: 31 days**

Whether the obtained fractions (Fr. M30 or Fr. M30-3-1) contain sex inducing substance can be checked by feeding them to asexual worms of the OH strain of *D. ryukyuensis* and whether they become sexual.

First, the fraction of interest is mixed with BSA and freeze-dried (1 day). The powder is then mixed with liver homogenate, freeze-dried again, and cut into 28 pieces (2 days). Finally, feeding assay is performed for 4 weeks (28 days).

BSA is mixed to make the freeze-dried powder visible and to prevent it from being scattered by static electricity. The planarians eat BSA without any problems. The absence of BSA does not affect the sex-inducing activity; we did not mix in the previous study.^1^

Unless specified, all steps should be performed at 20°C–23°C except for steps 64–66; steps 64–66 should be performed at 20°C.

***Note:*** We recommend checking the activity of other fractions eluting before and after the fraction of interest, just in case that the elution time of the sex-inducing substance may shift.

58. Freeze-dry the fraction of interest.
  a. Remove the fraction of interest from −80°C, thaw, and keep it on ice.
    ***Note:*** The fraction (e.g., 300 mL Fr. M30; 60 mL Fr. M30-3-1) must be concentrated to about 1–3 mL while removing the contained organic solvent with a rotary evaporator.
  b. Divide the required amount into, for example, a 25 mL conical centrifuge tube.
    ***Note:*** The obtained fractions were from 8 g of flatworms, and usually those from 4 g are sufficient to see sex-inducing activity.
  c. Add 1 mL of 1 mg/mL BSA solution and mix well by tapping (1 mg BSA per 150 μL of liver homogenate).
    ***Note:*** Fr. M30 does not need to be mixed with BSA since it is a sufficient amount of powder.
  d. Freeze at −80°C.
  e. Lyophilize as described in steps 8–9 (takes 6–12 h) (Figure 11A).

**Figure 11:**
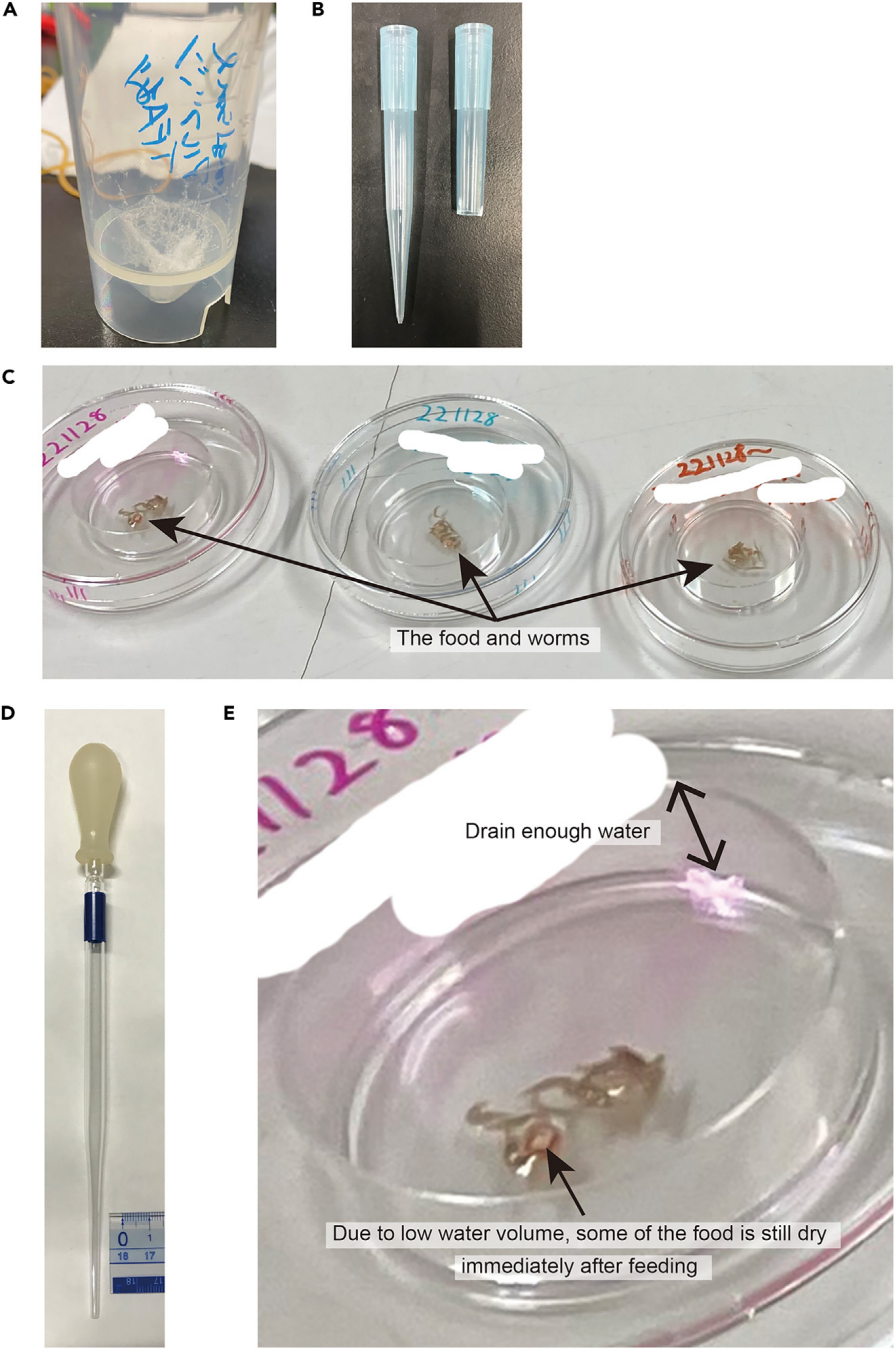
Feeding bioassay (A) Lyophilized powder of mixture of BSA and fraction of interest. (B) Cutting position of the 1000 µL tip. (C) The food and worms in a 35 mm plastic petri dish. (D) A glass pipette used for feeding bioassays. (E) Enlarged image of the petri dish during feeding bioassay.
59. Calculate the amount of liver homogenate needed for the feeding bioassay, in which 150 μL of the liver homogenate is used for one group of 15 test worms; for checking the activity of X fractions (including one control), 150X μL is needed.
60. Make liver homogenate.
  a. Freeze about 30 g of organic (i.e., hormone- and antibiotic-free) chicken liver.
    ***Note:*** Prepare at least 30 g, even if only a small amount is needed for the bioassay, as much is lost during homogenization. Excess liver homogenate can be stored in small portions at −80°C for 6 months, after which it will lose its taste and the planaria will no longer prefer it. Limit the number of freeze-thaw cycles to two or three.
  b. Remove the frozen liver from −80°C, place it in a 90 mm plastic petri dish, and slightly thaw it on ice.
  c. While frozen, chop it as finely as possible with scissors while keeping it on ice. Remove as much fat and muscle as possible at this stage.
    ***Note:*** Do not thaw it completely; otherwise, it will be difficult to chop.
  d. Homogenize the finely chopped liver further with 20 strokes, for example, using a Potter-type 10 mL homogenizer (Figure 4A).
    ***Note:*** Work while cooling with ice as heat is generated (Figure 4B).
    **CRITICAL:** If there are clumps in the liver homogenate, the freeze-dried sample powder will not mix evenly, so it must be homogenized completely. It will take a lot of force due to the high viscosity, but please be patient.
    **CRITICAL:** Do not add water. The freeze-dried food (including the sex-inducing substance) will get easily scattered in water during feeding.
  e. Transfer to a 25 mL conical centrifuge tube by decantation and keep it on ice until use. Since it is quite viscous and will remain on the homogenize tube walls, collect it as much as possible with a spatula.
  f. Repeat the process until the necessary amount is obtained.
61. Mix the powder (the fraction of interest) into the liver homogenate.
  a. Cut off the tip of a 1000 μL tip (Figure 11B), pipet the liver homogenate several times with a P1000 micropipette set to 150 μL, take 150 μL of the homogenate, and gently place it into a 25 mL conical centrifuge tube containing freeze-dried powder of the fraction of interest.
    ***Note:*** Do not let the tip touch the powder.
  b. Using a 200 μL tip, mix the liver homogenate and powder well on ice.
  c. After a brief flash spin, freeze at −80°C for 4 h.
    **CRITICAL:** A brief flash spin is recommended since strong centrifugal force may separate components (e.g., water). Ignore those adhering to the tube wall that still do not fall off.
62. Freeze-dry it as described in steps 8–9 for 6–12 h.
63. Prepare the food for 28 days of feeding bioassay.
  a. Place the freeze-dried food in a clean 90 mm plastic petri dish and cut into 28 pieces using a sharp razor blade (e.g., ultrathin carbon steel blade FA-10, FEATHER Safety Razor, Japan).
    ***Note:*** Small crumbs are produced, but continue cutting. To prevent small scraps, the tip is to insert the blade halfway through the food, tilt the blade slightly, and let the food break naturally.
    ***Note:*** It is not necessary to make the feed exactly even. Worms at the start of the bioassay are small and eat little, but they will grow and eat more during the bioassay. Feeding in the order of smallest to largest pieces ensures that the amount of food provided is appropriate for the body size of the worms.
  b. Place the pieces one by one into 0.5 mL microcentrifuge tubes (total 28 tubes).
    ***Note:*** Ignore the powdery stuff (probably planarians will not eat it), but collect any fragments that can be picked up with tweezers. If the pieces are too small, two pieces are combined for one day.
  c. Arrange the pieces in order of decreasing size and assign numbers so that the smallest pieces will be fed first.
  d. Store at −80°C.
64. Perform feeding bioassay at 20°C. Troubleshooting 1 and 2.
  a. Remove one tube with food from −80°C and bring it to 20°C (about 5 min).
  b. Collect 15 worms in a 35 mm plastic petri dish (Figure 11C).
  c. Drain enough rearing water with a glass pipette (Figure 11D) to prevent the food from floating (do not suck the worms) (Figure 11E).
  d. Place the food directly from the tube in the center of the petri dish (Figure 11C, arrow) by lightly popping the tube with your finger.
    ***Note:*** After a short time (2–3 min) after the rearing water is drained, the planarians settle down and begin circling around the petri dish. It is better to place the food in the center of the petri dish in this state to ensure equal access to the food.
  e. The worms will finish feeding in 10–20 min. Transfer the worms to a 90 mm plastic petri dish and change the rearing water several times to wash out food scraps.
  f. Transfer and maintain the planarians at a density of 5 worms per 90 mm plastic petri dish.
    ***Note:*** Daily feeding gradually increases body size, but the OH strain of *D. ryukyuensis* seldom does transverse fission at this density.
  g. Perform steps a–f once daily for 4 weeks (28 days).
    **CRITICAL:** The feeding should be performed at fixed times (±2 h) daily, maintaining a 24-h feeding interval to keep the worms' appetite.
65. After the last feed of the 4-week feeding bioassay, observe the worms under a microscope the next day.
  a. Gently place a worm in, for example, a plastic petri dish with a paintbrush. Rearing water should be limited to just keep the worm from drying out. At this point, the worm should settle into a position such that its ventral side adheres to the petri dish.
  b. Turn the petri dish over and observe the worm from the ventral side under a microscope. It is recommended to use a black background and illuminate from above at an angle with an external flexible arm light.

**Figure 12:**
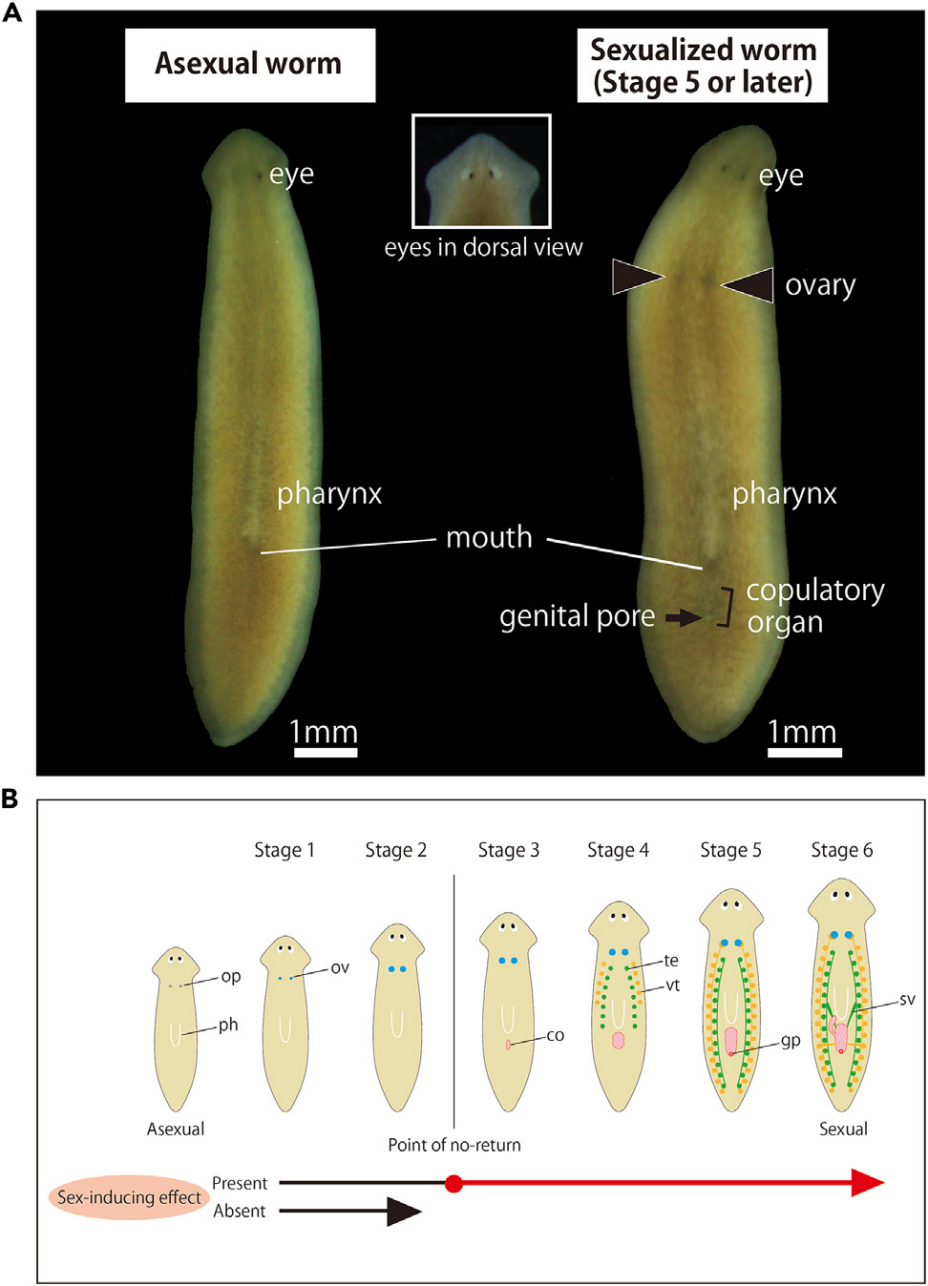
The sexualization of *D. ryukyuensis* (A) Images of asexual and sexualized (stage 5 or later) worms of the OH strain. Arrowhead indicates the ovary, the line indicates the copulatory organ, and the arrow indicates the genital pore. The worms are observed from their ventral side. Anterior side is up and posterior side is down. (B) Six stages of sexualization in the planarian *Dugesia ryukyuensis*. The worm begins to develop hermaphroditic reproductive organs upon switching from the asexual to the sexual state. The asexual worm possesses the ovarian primordia (op); during stage 1, the number of oogonia increases in the ovary (ov); during stage 2, the ovary begins to mature and develop oocytes; during stage 3, the copulatory organ (co) begins to form; during stage 4, the primordial testis (te) and primordial vitellaria (vt)^17^ begin to form; during stage 5, the genital pore (gp) becomes externally apparent; during stage 6, the worm becomes sexually mature with well-developed seminal vesicles (sv), ready for mating and egg-laying. Between stages 2 and 3, there is a point of no return, marking the developmental phase from which the sexualization process proceeds without further administration of sex-inducing substances. The sex-inducing effects were evaluated based on whether the OH strain of the planarian *D. ryukyuensis* changed beyond the point of no return in a feeding bioassay. Note that the increase in planarian body size is due to the feeding bioassay procedure: if a food does not contain sex-inducing substances, asexual worms become bigger without gonadal development. ph, pharynx. This figure is part of figures from Sekii et al. (2023).^1^

***Note:*** The criteria to distinguish the ventral from dorsal sides are as follows. On the ventral side, the mouth (i.e., the pore from which the pharynx comes out) is observed, and the genital pore is also observed below it in sexual individuals (Figure 12A). On the dorsal side, however, these pores cannot be observed, and instead the eyes are more clearly visible (Figure 12A).

***Note:*** No anesthetic solutions are used. If the worms move violently and are difficult to observe, cool the petri dish on ice to slow their movement (so that the ice does not directly touch the worms).

66. Sexualization progresses through six stages (Figure 12B).
  a. There is a point of no return between stages 2 and 3, beyond which sexualization proceeds irreversibly.
  b. The sex-inducing substance can make worms cross the point of no return. To confirm the presence of sex-inducing activity, the copulatory organ formed after stage 3 should be observed, besides the ovary.
  c. If "only" ovaries are visible, sex-inducing activity cannot be determined to be present. Troubleshooting 3, 4, and 5.

***Note:*** If sex-inducing activity is strong, the ovaries (Figure 12A, arrowhead) become visible about 2 weeks, and the copulatory organ (Figure 12A, indicated in a line) about 3 weeks after the start of the assay. However, it is difficult to observe immediately after feeding.

***Note:*** The observation of the copulatory organ is used as the criteria for being sexualized (i.e., beyond the point of no return) because testes and yolk glands cannot be observed externally under the microscope. To assess the formation of testes and yolk glands, histological observations using HE staining or quantitative RT-PCR for increased expression of marker genes should be performed.^1^

**CRITICAL:** If sexual maturity cannot be determined after the bioassay, the worms should be fed normal chicken liver once a week for another 4 weeks. If the fed fraction has sex-inducing activity, the worms have exceeded the point of no return^13^ (Figure 12B) during the feeding bioassay, and their sexualization will autonomously progress during the additional 4 weeks. The genital pore (Figure 12A, arrow) will be visible, which is another small pore below the mouth that is easier to observe.

## Expected outcomes

**Figure 13:**
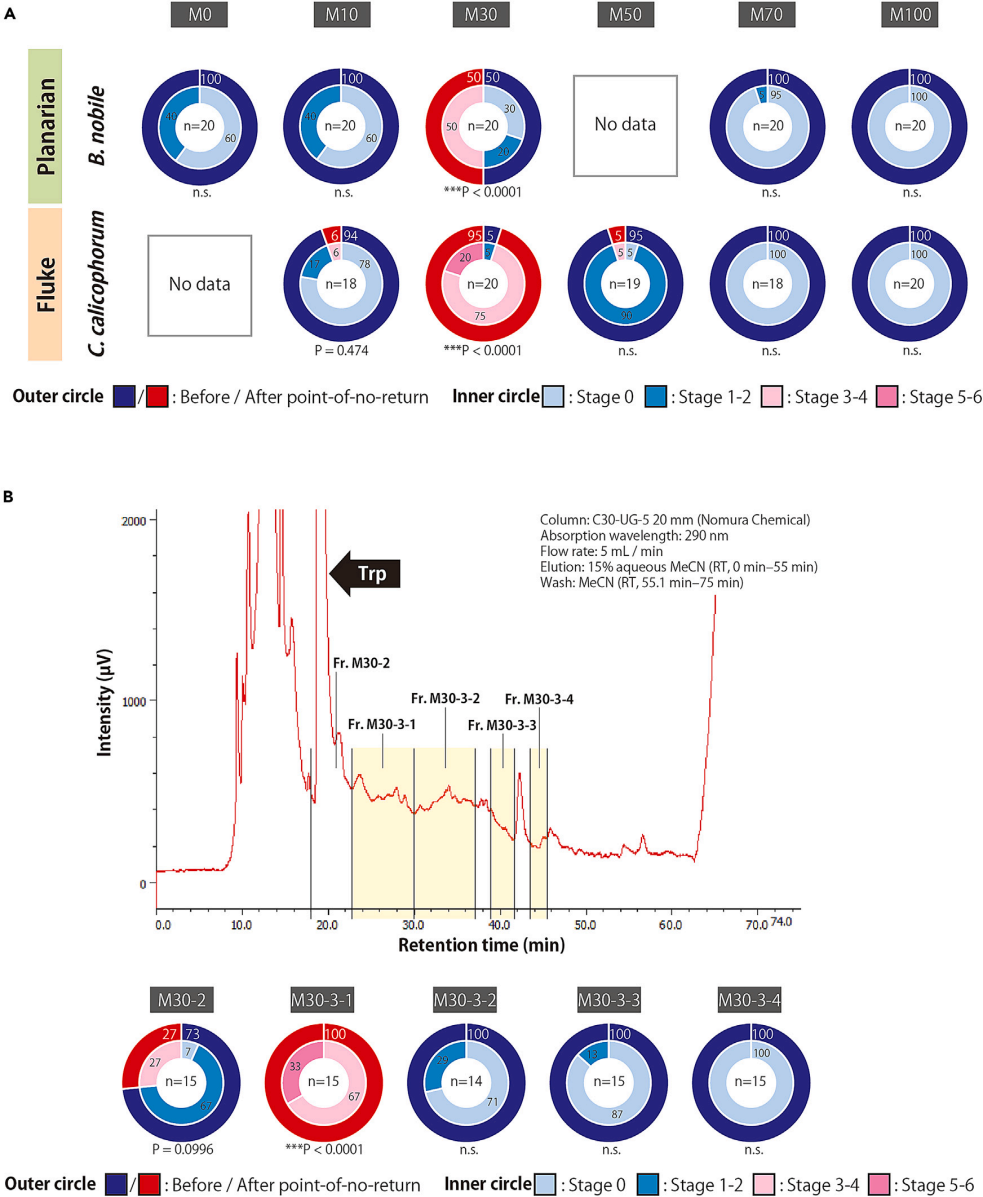
Expected outcomes for the sex-inducing activity of Fr. M30 and Fr. M30-3-1 (A) The sex-inducing effects of Frs. M0, M10, M30, M50, M70, and M100 on asexual *D. ryukyuensis* worms were examined using a feeding bioassay for 4 weeks. Samples derived from 4 g of *B. nobile* worms or 2 g of *C. calicophorum* worms were used in the feeding bioassays. (B) Fr. M30-3 indicated in yellow were further fractionated into four fractions via reverse-phase HPLC. Note that several peaks were observed in Frs. M30-3-1 and M30-3-2, but these peaks did not appear every time. Therefore, Frs. M30-3-1 and M30-3-2 were collected based on the retention time (approximately every 7 min) after the appearance of the tryptophan peak. The samples derived from 2 g of *C. calicophorum* worms were injected into the HPLC system via four separate injections. The sex-inducing effects of each fraction on asexual *D. ryukyuensis* worms were examined using a feeding bioassay for 4 weeks. The percentages of worms in different developmental states are presented in doughnut charts; the outer circle shows the worms before and after the point of no return, and the inner circle shows the sexualization stages of the worms. White and black numbers in the circles indicate percentages. Asterisks indicate significant differences in the number of worms before and after the point of no return between the control and focal groups (Fisher’s exact test: ∗∗∗p < 0.001; n.s., not significant). Source data and statistics, including the exact p-values, are available in Supplemental Dataset 5.^1^ The sample size of each group is shown in the center of the doughnut chart. These figures are part of figures from Sekii et al. (2023).^1^

As expected outcomes, we show the results of feeding bioassays of Frs. M0-M100 obtained by open-column chromatography using the terrestrial planarian *B. nobile* and the fluke *C. calicophorum* (Figure 13A); and the results of reverse-phase HPLC for the *C. calicophorum* Fr. M30 and of feeding bioassay of those fractions (Figure 13B). Both results were already reported by Sekii et al. (2023).^1^

## Limitations

A limitation of this protocol is that it is impossible to quantify the amount of sex-inducing substance in the obtained fraction because it is an unknown compound.

Another limitation is that the protocol requires the use of the OH strain of the planarian *D. ryukyuensis* for the feeding bioassay to determine if the extracted fraction contains the sex-inducing substance. Other planarian species or those caught in the field may be used for sexualization, but we do not recommend it for the following reasons: (1) Some of the field-caught planarians may spontaneously become sexualized with temperature changes or for other unknown reasons, in the absence of stimulation from a sex-inducing substance. Contrastingly, the OH strain never spontaneously sexualized in 40 years when kept at 20°C, making it a suitable test organism for evaluating the activity of the crude purified fraction that has been extracted. (2) Other planarian species may be less susceptible to the sex-inducing substance. In particular, planarians caught in the field have individual differences in sensitivity to the sex-inducing substance. Even under conditions in which 100% of the OH strain individuals would be sexualized, some planarians may not become sexualized when using individuals from a different strain.

For example, the asexual strain of the planarian *Schmidtea mediterranea* (CIW4 strain), which are used worldwide as an excellent model organism for stem cell biology, was impossible to be sexualized even after one month of feeding *B. brunnea* or *B. nobile*. These results were confirmed by histological observation. This is probably owing to chromosomal translocation^16^ in the CIW4 strain. Other asexual strains of *S. mediterranea* have not been tested. Further, the HI strain of *Dugesia japonica* was not sexualized by feeding alone and needed to be subjected to a cold stimulus. The GI strain of *D. japonica* can be sexualized, but the efficiency of the process is unstable and gradually decreases after several years of maintenance under laboratory conditions. The possibility that similar problems occur in individuals in the field cannot be ruled out. In this aspect, the OH strain can be stably sexualized by chemical stimulation with the sex-inducing substance, making it an easy option for evaluating the activity of the extracted fraction.

## Troubleshooting

### Problem 1

The plastic Petri dish used to feed repels rearing water, which is common with new petri dishes (Methods video S8) (related to Step 64).

Methods video S8. Troubleshooting for when a plastic petri dish repels rearing water, related to troubleshooting, problem 1

### Potential solution

• Drain rearing water slowly and stop at a point where too much water is not drained (Methods video S8).
• If the petri dish has repelled rearing water after the food has been added, add one drop of rearing water near the food and use surface tension to connect the water from around the food using the tip of tweezers.

### Problem 2

Planarians were not eating food during feeding bioassay (related to Step 64).

### Potential solution

• Ideally, they should start eating within 10 min, but it can take longer, so wait 30–40 min. Once someone starts eating, everyone tends to come closer.
• Too much rearing water has been drained, therefore add 2–3 drops of rearing water and observe what happens. If there is no change, add another 2–3 drops of rearing water after 5 min.
• Planarians do not like light, so the light should not be too bright. However, normal fluorescent light intensity for human work is not a problem. Refrain from showing them shadow movement by frequently looking into it.
• It is normal to have occasional days in four-week period when planarians do not eat. It is best to record the days when the food is left behind.
• Depending on the flatworms used, it cannot be ruled out that substances not preferred by the planarians may have been eluted together in the fraction of interest. If this is the case even with the highly purified Fr. M30-3-1, it is very unfortunate and we cannot advise a solution.

### Problem 3

Absence of sex-inducing activity in the tested fraction (i.e., the planarians are not sexualized even though they are feeding properly) (related to Step 66).

### Potential solution

• Check the activity of other fractions (e.g., Frs. M10 and M50, and fractions eluting before or after the reference time), as the retention time of the sex-inducing substance may have shifted and eluted into other fractions. For example, (1) changes in the pH of the sample may change the elution time. In the case of the planarian *B. nobile*, the pH of the sample before open-column chromatography was approximately 6.5, measured with pH test paper. (2) Temperature can also affect elution time, so check for any factors that may cause temperature instability during fractionation. (3) As the retention time of substances may vary depending on the HPLC system, check the retention time of tryptophan, which represents a reproducible landmark peak for estimating the retention time of the sex-inducing substance, and compare it with that obtained with our HPLC system (Figure 13B).
• Run HPLC several times with tryptophan solution to check for the reproducibility of peak positions.

### Problem 4

Absence of sex-inducing activity in all fractions (related to Step 66).

### Potential solution

• Review the precautions for the flatworms to be used again (described in the “before you begin” section). If the flatworm meets the requirements, feed it as it is and check its activity. This is the most efficient method of administering the sex-inducing substance because of minimal loss due to purification. If the activity is still not confirmed, it is possible that the flatworms used do not contain the sex-inducing substance (if this is the case, please let us know by email, as it is very interesting).

### Problem 5

Difficulty in observing the ovary, copulatory organ, and genital pore (related to Step 66).

### Potential solution

• Changing the angle of illumination.

## Resource availability

### Lead contact

Further information and requests for resources and reagents should be directed to and will be fulfilled by the lead contact, Kazuya Kobayashi (kobkyram@hirosaki-u.ac.jp).

### Materials availability

• This study did not generate new unique reagents.
• The OH strain of the planarian *Dugesia ryukyuensis* is available on the request.

## Data Availability

This study did not generate/analyze datasets/code.
